# Endoperoxide delivered singlet oxygen: the future of PDT, without light or oxygen

**DOI:** 10.1039/d5md00887e

**Published:** 2026-01-17

**Authors:** Wanwan Wang, Lei Wang, Rensong Sun, Xiao Qian, Ziang Liu, Engin U. Akkaya

**Affiliations:** a State Key Laboratory of Fine Chemicals, Department of Pharmaceutical Engineering, Dalian University of Technology 116024 Dalian P. R. China leiwang@dlut.edu.cn eua@dlut.edu.cn; b Department of Chemistry, Bilkent University 06800 Ankara Turkey

## Abstract

Chemically generated singlet oxygen *via* cycloreversion reaction of aromatic endoperoxides is poised to evolve into a highly promising therapeutic protocol. Singlet oxygen can also be produced endogenically, with a short half-life especially in biological media, and it acts locally, only when a threshold value is exceeded. Conserving the essence of photodynamic therapy, which is the delivery of singlet oxygen to tumors, two limiting issues of light penetration and low tumor oxygenation can be circumvented simultaneously by endoperoxide-delivered singlet oxygen. The endoperoxides are also amenable to derivatization for more specific targeting as well. In this work, pyridone-endoperoxides with mitochondria targeting triphenylphosphonium moieties were shown to target tumors and result in significant tumor suppression. The series of endoperoxides tested also confirms the importance of mitochondria targeting. In mouse tumor models, these compounds show no signs of systemic or organ level toxicity.

## Abstract 2

Chemically generated singlet oxygen *via* cycloreversion reaction of aromatic endoperoxides is poised to evolve into a highly promising therapeutic protocol.

## Graphical abstract

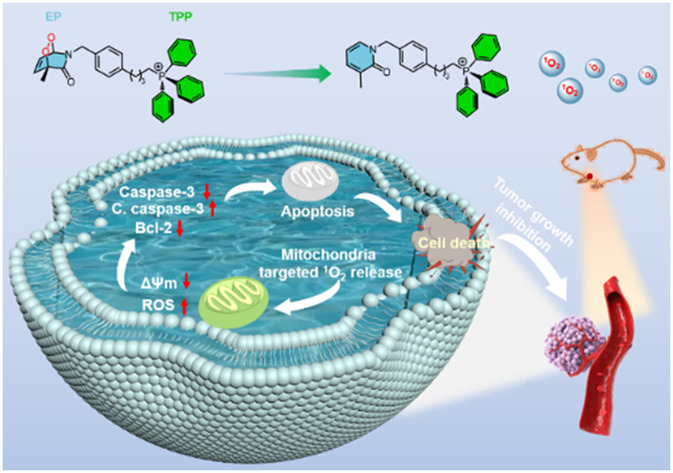

## 1. Introduction

The “photodynamic effect”, first defined more than 125 years ago,^1^ was later understood to be photosensitized generation of singlet oxygen through the intermediacy of an organic dye (photosensitizer). This effect has evolved into a cancer therapy modality (photodynamic therapy, PDT) following the clinical studies started at Roswell Park Memorial Institute in the late 1970s.^2^ The FDA approved the photosensitizer Photofrin® for palliative treatment of advanced esophageal cancer in 1994,^3^ and today, it is approved for the treatment of specific lung cancer types and Barrett's esophagus.^4^ However, over the years, it has become clear that the very limited light penetration through tissues,^5^ even at optimal wavelengths^6^ (a.k.a., therapeutic window), will confine PDT to be a superficial treatment, most appropriate for certain skin conditions, including melanoma, and not a broadly-applicable first-line therapy.^7^

Recent research activity regarding various modifications or improvements to PDT seems to disregard the light penetration issue and the huge difference in the relevance of light penetration in a mouse model *versus* a potential human patient^8^ considering many variances, most obviously the difference in size.

On the other hand, we previously proposed^9,10^ the idea that endoperoxides can be utilized as singlet oxygen delivery agents under certain conditions, for therapeutic purposes, thus eliminating the need for external photonic excitation.^10^ If the delivery^11^ is selective enough, this would be a significant development transforming PDT into a special therapeutic protocol, distinct from chemotherapy, because the carrier of singlet oxygen,^12,13^ or the metastable “storage compound” needs not to be toxic at all, and the anti-tumor activity should be only from the released singlet oxygen. Endoperoxide generated singlet oxygen was studied for its cytotoxic potential a few decades ago,^14^ but the endoperoxides used in those studies were negatively charged and were not likely to be cell permeable. Also, the IC_50_ values against HepG2 cancer cells were reported to be >5 mM. These early discouraging results were probably the reason for the lack of further interest in this line of research. Another reason is the correct, but misleading categorization that photosensitized generation of singlet oxygen is a catalytic process, whereas singlet oxygen release from endoperoxides is obviously stoichiometric. This may lead to the incorrect assumption that at any reasonable dosage of endoperoxides, the singlet oxygen released would not be sufficient to cause cellular death in tumors. However, a calculation of the threshold doses of singlet oxygen (generated by photosensitization) required for reducing the survival of cancer cells to 1/*e* yielded 3.6–4.7 × 10^7^ singlet oxygen molecules per cell *in vivo* (in a radiation-induced fibrosarcoma tumor mouse model).^15^ Even allowing for large error margins in the calculations, the amount is easily attainable by endoperoxide cycloreversion reactions *in vivo*.

Recently, in our research group, we have been focused to explore^16–19^ various aspects of controlling singlet oxygen release in cell cultures and *in vivo*. The results displayed significant potential for therapeutic utility and suggested that targeting mitochondria may increase the efficacy of the endoperoxide delivered singlet oxygen approach.

## 2. Results and discussion

**Fig. 1:**
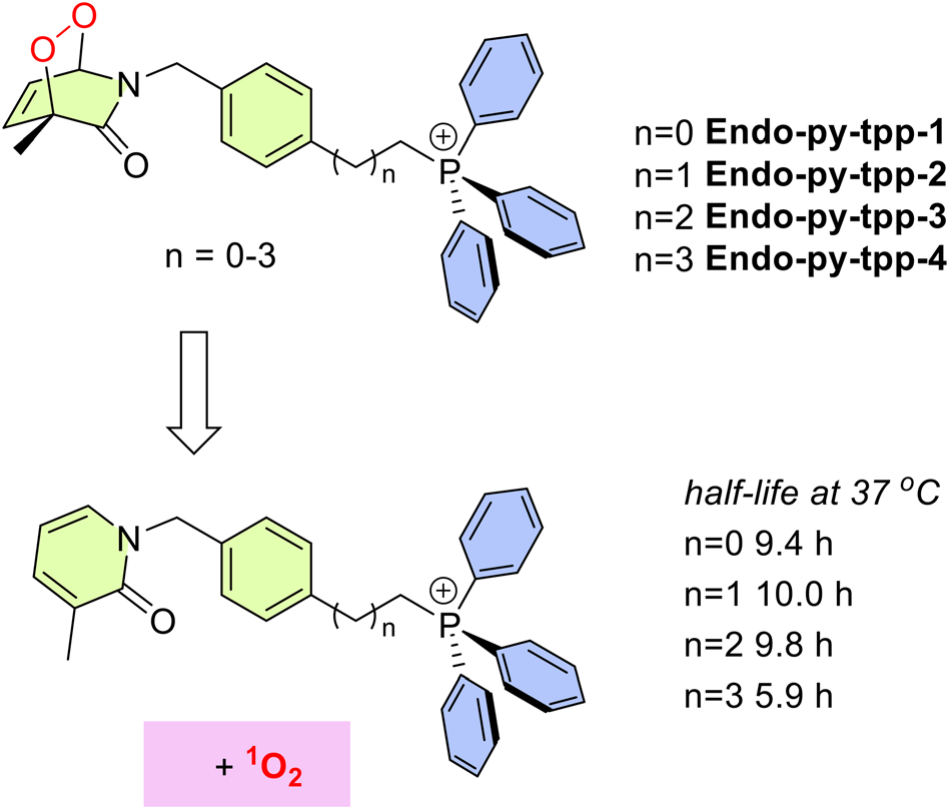
General structures of mitochondria targeting singlet oxygen sources synthesized and studied in this work. Half-lives for cycloreversion reactions were determined in CDCl_3_.

**Fig. 2:**
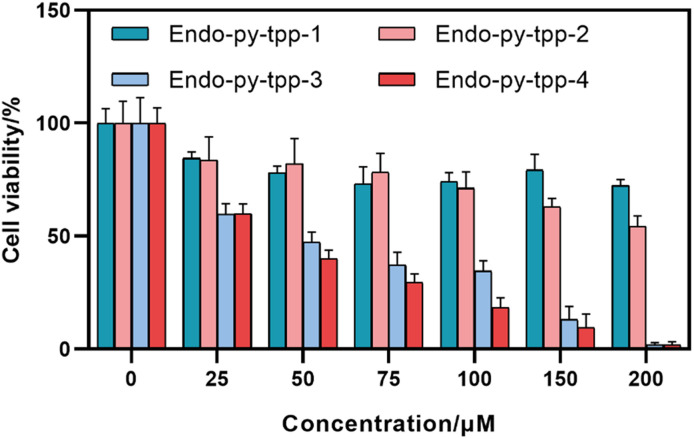
Cell viability of A549 cells treated with different endoperoxides at indicated concentrations.

In order to clarify the role of mitochondria targeting *in vivo*, in this work, we targeted the synthesis (SI) of a series of 2-pyridone endoperoxides (Fig. 1) with a triphenylphosphosphonium (TPP) module. The half-lives for the first order cycloreversion reactions were determined by ^1^H NMR spectroscopy by following the changes of integrals for the well-resolved characteristic peaks of the endoperoxide and pyridone, such as the methyl substituent on the pyridone ring. As the reaction proceeds, the singlet at 1.61 ppm is replaced by another singlet at 2.11 (SI, Fig. S1–S4). The half-lives of endoperoxide cycloreversion reactions are around 10 h at 37 °C, however with larger distance from the TPP unit and less steric hindrance, **Endo-py-tpp-4** decays a little faster. We then carried out MTT assays to assess the cytotoxicity of these compounds using various cancer cell lines. IC_50_ values range between 30 and 60 μM for the longest linker (Table 1), and it is clear that, if mitochondria cannot be targeted, the release of singlet oxygen is much less effective. Short chain linkers apparently do not allow transport through mitochondrial membranes as evidenced by large IC_50_ values (>200 μM). This is in agreement with previously reported data using non-targeted endoperoxides (Fig. 2).^10,14^

**Table 1:** IC_50_ values (μM) of **Endo-py-tpp-3** and **Endo-py-tpp-4**

Compound	A549	MCF-7	4T1	HeLa	HepG2
**Endo-py-tpp-1**	>200	>200	>200	>200	>200
**Endo-py-tpp-2**	>200	>200	>200	>200	>200
**Endo-py-tpp-3**	58.52	67.79	108.3	89.08	80.24
**Endo-py-tpp-4**	46.43	58.99	76.74	82.45	77.02

**Fig. 3:**
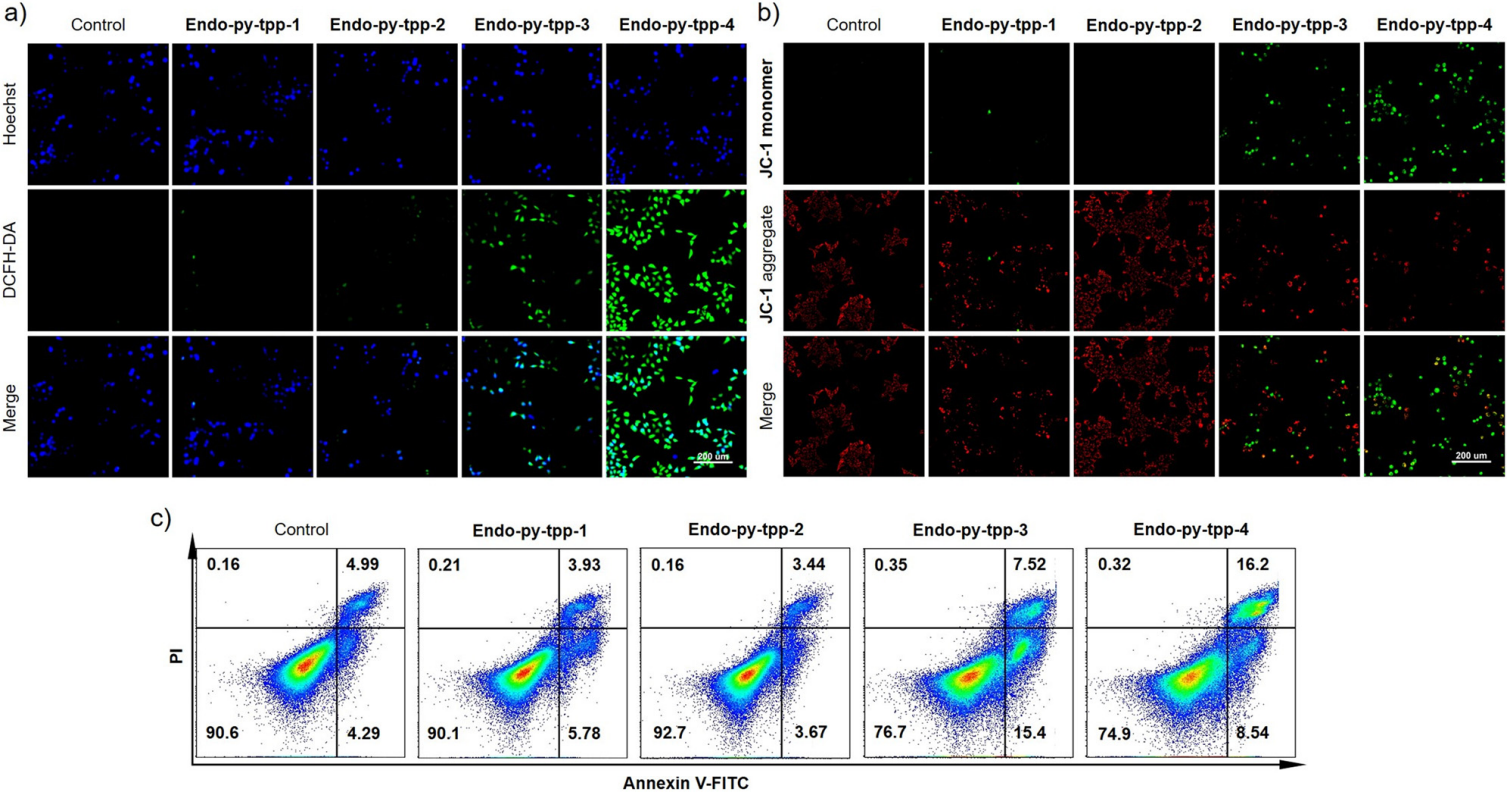
a) Detection of intracellular ROS after treatment with different groups, 40 mM; b) JC-1 staining with different treatments. A549 cell culture was used; c) flow cytometry analysis of apoptosis after different treatments.

The compounds were tested for singlet oxygen release using the probe compound DPBF in DMF and in PBS buffer at pH 7.2, using singlet oxygen selective fluorescent probe SOSG. The results qualitatively corroborate ^1^H NMR data regarding singlet oxygen release from all four compounds, with **Endo-py-ttp-4** being slightly faster (Fig. S9–S22). Stock solutions were prepared in DMSO, never to exceed 1% in the final experiments. Fluorescence microscopy by double staining with Hoechst nuclear stain and DCFH-DA (ROS probe) showed the different outcomes due to the linker length difference. **Endo-py-ttp-1** and **Endo-py-ttp-2** seem not to be cell-permeable, or not to localize in mitochondria and wash out too quickly to react with the fluorescent probe. Characteristic green fluorescence, indicative of released singlet oxygen, was only observed (Fig. 3a) in the cells (A549 cell line) incubated with **Endo-py-ttp-3** and **Endo-py-ttp-4**. Selective probe Si-DMA, which reports only mitochondrial singlet oxygen, shows a similar picture; again only the two longer alkyl chain endoperoxides release singlet oxygen inside the mitochondria (Fig. S23).

It is well known that apoptosis^20^ is closely linked to the collapse of mitochondrial membrane potential (Δ*ψ*_m_). The cationic fluorescent probe, JC-1 (5,5,6,6′-tetrachloro-1,1′,3,3′-tetraethylbenzimidazoylcarbocyanine iodide), is very specific for measuring changes in Δ*ψ*_m_. In the mitochondria of normally functioning cells, JC-1 forms J-aggregates with red emission, but when membrane potential is lost, aggregates unravel, and green monomer emission is observed. Staining the cancer cells (A549) with this dye revealed loss of mitochondrial membrane potential only with **Endo-py-ttp-3** and **Endo-py-ttp-4** (Fig. 3b).

The flow cytometry results of cells incubated with the endoperoxides also display a significant increase in apoptosis in the longer linker conjugates (Fig. 3c).

**Fig. 4:**
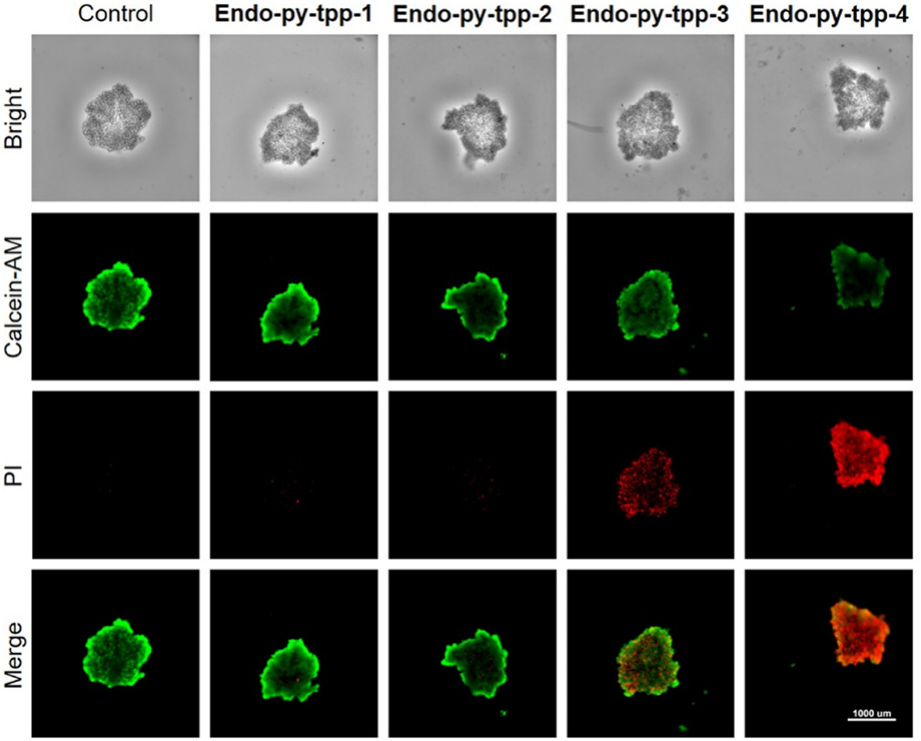
A calcein-AM/PI apoptosis detection kit was used for assessing endoperoxide-induced apoptosis in multicellular tumor spheroids.

Additional high content imaging of cells with live-dead assays using calcein-AM and PI dyes shows a larger fraction of A549 cell death under incubation with **Endo-py-ttp-4** (Fig. S25). Along the same lines, we used A549 cells to generate tumor spheroids from single-cell suspensions. A similar staining procedure after incubation with the endoperoxides confirms the effectiveness of **Endo-py-ttp-4** in 3D cell cultures (Fig. 4) as well.

**Fig. 5:**
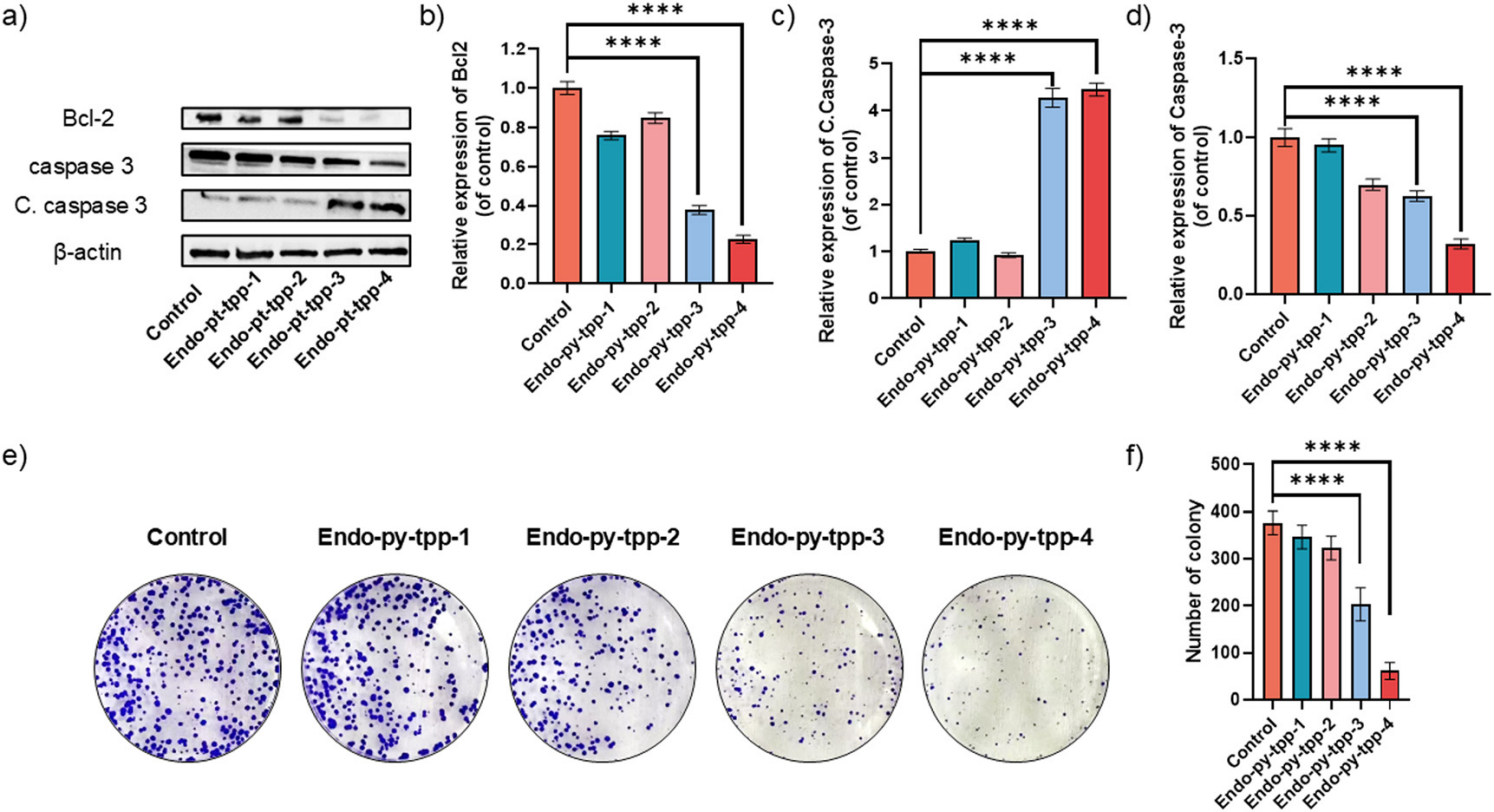
a) Expression levels of apoptosis related proteins, b) relative expression of Bcl-2 under different treatments, b) relative expression of caspase-3 under different treatments, and c) relative amount of cleaved caspase-3 under different treatments. e) Colony formation assay experiments and f) the number of colonies with different endoperoxide treatments. All experiments were carried out with A549 cells. Data are shown as the mean ± SD, *n* = 3 per treatment. ****p < 0.0001.

Following these experiments, we wanted to analyze changes in the expression of critical proteins linked to the apoptotic process using western blot analysis. A549 cells were lysed after treatment with the endoperoxides. The expression levels of proteins Bcl-2 and caspase 3 and the amount of cleaved caspase 3 were studied. Bcl-2 is an integral outer mitochondrial membrane protein that blocks apoptotic death. Our data show that Bcl-2 expression is significantly decreased in the cells treated with **Endo-py-ttp-3** and **Endo-py-ttp-4**. Also, caspase-3 levels decrease, while cleaved caspase-3 levels increase significantly (Fig. 5a–5d).

We also studied the effect of singlet oxygen releasing pyridine endoperoxides by colony formation assays as they provide clear information about their cellular clonogenic potential. The data clearly confirm the superiority of **Endo-py-ttp-4** in inhibiting colony formation of A549 cells.

Scratch tests, also known as cell migration assays, were conducted (Fig. S26). The ability of cells to migrate is essential in tumor invasion, neoangiogenesis and metastasis.^21^**Endo-py-ttp-4** is particularly effective in inhibiting cancer cell migration.

**Fig. 6:**
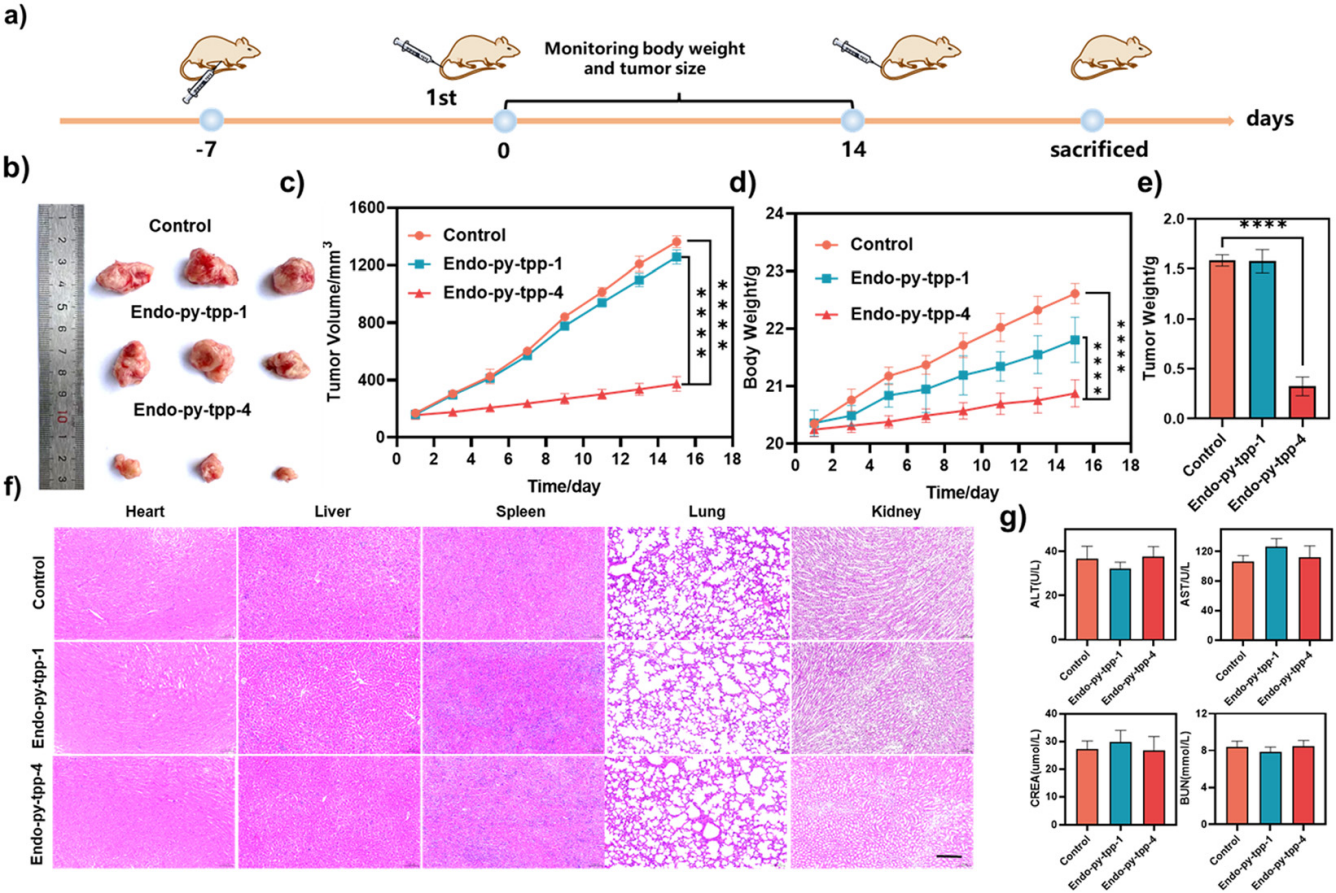
a) Schematic of the experimental protocol. b) Representative tumor images after different treatments. c) Inhibition of 4T1 tumor volume in different groups. d) Time evolution of mouse weight under different treatments. e) 4T1 tumor weight under different treatments. f) H&E staining of different organs in various groups of mice. Scale bar: 100 μm. g) Blood biochemistry analysis of the mice after the treatment of **Endo-py-tpp-1** and **Endo-py-tpp-4** including alanine transferase (ALT), aspartate transaminase (AST), creatinine (CREA), and blood urea nitrogen (BUN). Data are shown as the mean ± SD, *n* = 3 per treatment. ****p < 0.0001.

Encouraged by these results, we initiated *in vivo* experiments. The tumor model was developed by injecting 4T1 cells subcutaneously into the armpit of 5–6 week old female BALB/c mice (Fig. 6a). The tumor-bearing mouse models were raised for approximately 8 days, and then divided into three groups (five mice in each group): the first group was the control group, the second group was treated with **Endo-py-tpp-1** (14 mg kg^−1^), and the third group was treated with **Endo-py-tpp-4** (15 mg kg^−1^). The results show that **Endo-py-tpp-4** was very effective (Fig. 6b–e) in suppressing tumor growth (the average tumor mass was 1.55 g in the control group and 0.4 g in the **Endo-py-tpp-4** group). The body weight loss observed in mice treated with endoperoxides was within the acceptable limits (<10%). H&E staining showed no abnormal changes in major organs (Fig. 6f) and blood enzyme levels including alanine transferase (ALT), aspartate transaminase (AST), creatinine (CREA), and blood urea nitrogen (BUN) (Fig. 6g), confirming the systemic safety of the treatment protocol. Representative images from H&E and Ki67 staining of tumor tissues showed remarkable changes in the tumor appearance and characteristics. Treated tumor tissues show minimal Ki67 staining, which is a clear sign of inhibited mitosis (Fig. S27).

## Conclusion

In summary, successful targeting of mitochondria by singlet oxygen releasing pyridine endoperoxides shows significant potential in cancer therapy without any dependence on oxygen or light. This may be the way forward in transforming PDT into a more clinically accessible protocol by essentially keeping the essence of its action and cutting its dependence on external excitation, which cannot be satisfactorily provided for most tumors. In other words, as long as external excitation in the visible-near IR region is required for any therapeutic protocol for cancer (PDT, PTT, *etc.*), the methodology is very likely to be limited to superficial tumors at best. This realization should cause a significant change in the research direction of the PDT community at large. We will continue to explore this new path in our own research program.

## Author contributions

E. U. A. directed the project and conceived the idea and designed the initial strategy. W. W. and L. W. designed the experiments, evaluated the data and wrote the first version of the manuscript. E. U. A. wrote the final version of the manuscript. R. S., X. Q. and Z. L. assisted with the synthesis and characterization of the compounds.

## Conflicts of interest

There are no conflicts to declare.

## Supplementary Material

MD-017-D5MD00887E-s001

## Data Availability

The data supporting this article have been included as part of the supplementary information (SI). Supplementary information: synthesis procedures, additional characterization data. See DOI: https://doi.org/10.1039/d5md00887e.
