# Supplementary material for: Endoperoxide delivered singlet oxygen: the future of PDT, without light or oxygen
Source: RSC Med Chem. 2026 Jan 17;17(3):1729–34. doi: 10.1039/d5md00887e (PMC12946930; doi:10.1039/d5md00887e)
Supplement: MD-017-D5MD00887E-s001 [file MD-017-D5MD00887E-s001.pdf]

Supplementary Information (SI) for RSC Medicinal Chemistry.
This journal is © The Royal Society of Chemistry 2026

Table of Contents

1. Experimental Section 2

2. Synthesis of endoperoxides 3

3. Temporal Evolution of endoperoxides ^{1}H NMR 12

4. Detection of singlet oxygen release 18

5. MTT assay 26

6. Detection of singlet oxygen for intracellular generation 27

7. Detection of mitochondrial membrane potential 28

8. Detection of singlet oxygen within mitochondria 29

9. Cell apoptosis experiment 30

10. Live/dead cell staining using Calcein-AM/PI 31

11. 3D (three-dimensional) tumourspheres 32

12. Western blot 33

13. Colony formation assay 34

14. Wound healing scratch assay 35

15. Establishment of tumor model 36

16. NMR Spectra 37

1. Experimental Section.

Materials: Unless otherwise specified, all reagents and solvents were purchased from commercial suppliers and used without further purification. Reactions were monitored by thin layer chromatography using Huang-hai TLC Silica gel 60 F-254. Column chromatography was performed by using Mei-gao Silica Gel 60 (particle size: 200-300 mesh). 1,3-Diphenylisobenzofuran (DPBF) was purchased from Shanghai aladdin Co., Ltd (Shanghai, China). Singlet Oxygen Sensor Green (SOSG) was purchased from Thermo Fisher Scientific Inc. (Shanghai, China). Reactive Oxygen Species Assay Kit and Calcein-AM/PI Live/Dead Cell Double Stain Kit was bought from Beijing Solarbio Science & Technology Co., Ltd (Beijing, China). DAPI staining solution and Annexin V-FITC Apoptosis Detection Kit were bought from Shanghai Beyotime Biotechnology Co., Ltd (Shanghai, China).

Instruments: The ^{1}H and ^{13}C NMR spectra were recorded using Bruker Vaian DLG400. Chemical shifts were reported in parts per million (ppm) and coupling constants (*J* values) are given in Hz. Splitting patterns are indicated as follow s, singlet; d, doublet; t, triplet; m, multiplet. The UV-Vis absorption spectra were performed by using Agilent Cary-3500 UV-Vis spectrophotometer. All animal experiments described in this paper were approved by the Animal Ethics Committee of Dalian University of Technology, ethics approval number is DUTSCE250318-01.

2. Synthesis of endoperoxides

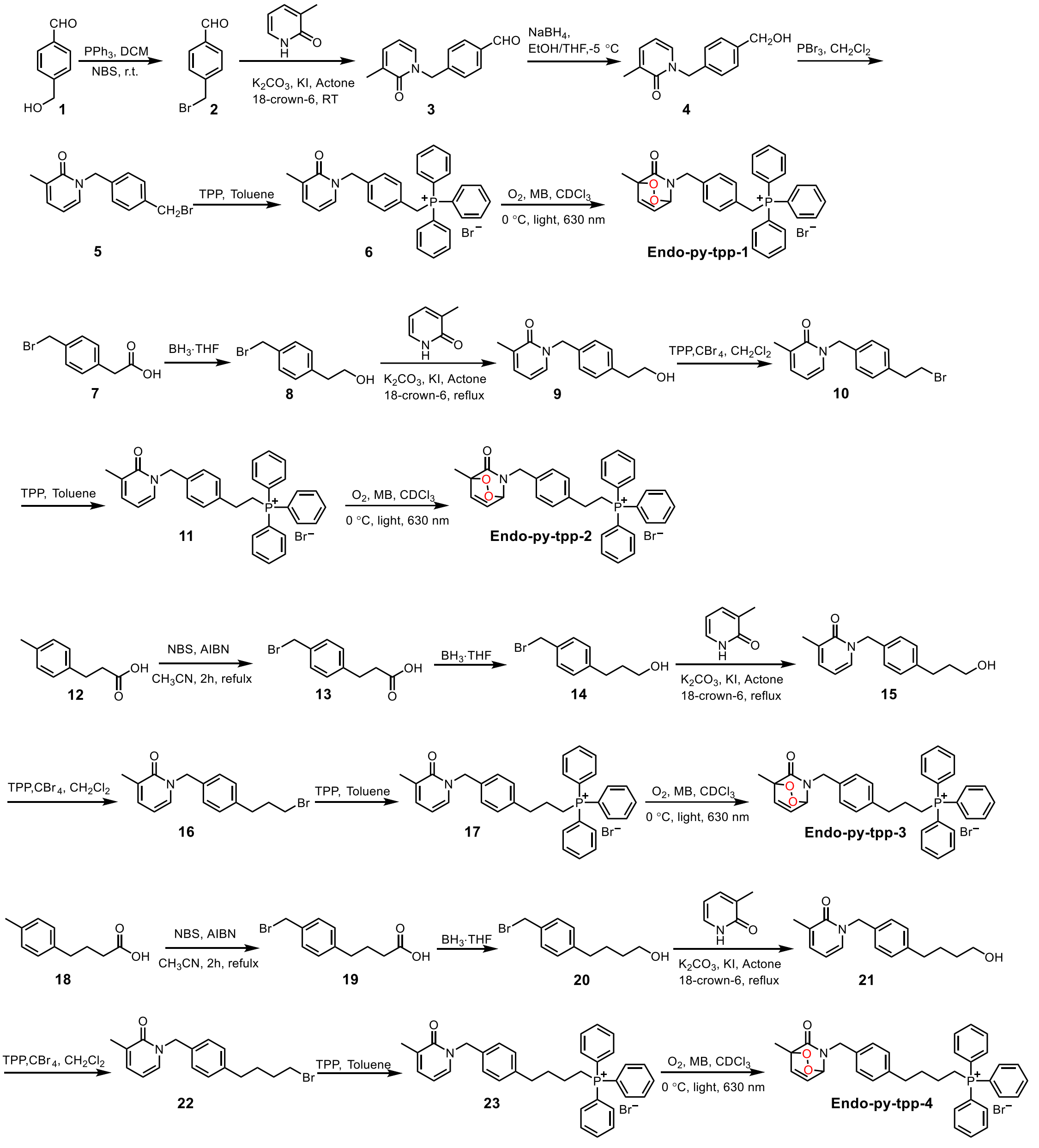

Scheme S1. The synthesis of endoperoxides.

General Comments about the endoperoxide synthesis:

Atmost attention was paid during the final endoperoxide synthesis steps to keep the endoperoxides at cold temperatures. Reaction was carried out at 0 ºC and the column chromatography was done using a jacketed-chromatography column with 4 ºC coolant

circulating. The solvent used was CH_2Cl_2/Methanol (15 : 1, v/v). The fractions collected were kept in ice.

*Synthesis of **2***: PPh_3 (574 mg, 2 mmol), Compound **1** dissolved (272 mg, 2 mmol) in 20.0 mL DCM at room temperature. Then N-bromosuccinimide (NBS) was added to the mixture slowly. The progress of the reaction was monitored by TLC. When the reaction was completed, concentrated under vacuo. The residue was purified by silica gel flash column chromatography to get a white powder (293 mg, 75%).^{1}H NMR (400 MHz, Chloroform-*d*) δ 9.89 (s, 1H), 7.73 (d, J = 7.9 Hz, 2H), 7.43 (d, J = 7.9 Hz, 2H), 4.40 (s, 2H). ^{13}C NMR (101 MHz, $CDCl_3$) δ 191.43, 144.16, 136.02, 130.06, 129.62, 32.07.

*Synthesis of **3***: K_2CO_3 (0.7 g, 2.07 mmol), compound **2** (400 mg, 2.02 mmol) and 3-methyl-2-pyridone (185 mg, 1.69mmol) dissolved in acetone. After adding catalytic amount of 18-benzocrown-6 and KI, reaction was left to stir at room temperature. The progress of the reaction was monitored by TLC. When the reaction was completed, concentrated under vacuo. The residue was purified by silica gel flash column chromatography to get a white powder (410 mg, 89%). ^{1}H NMR (400 MHz, Chloroform-*d*) δ 9.89 (s, 1H), 7.77 (d, J = 7.8 Hz, 2H), 7.37 (d, J = 7.8 Hz, 2H), 7.16 (d, J = 6.8 Hz, 2H), 6.06 (t, J = 6.8 Hz, 1H), 5.14 (s, 2H), 2.09 (s, 3H). ^{13}C NMR (101 MHz, $CDCl_3$) δ 191.68, 162.85, 143.43, 137.02, 135.74, 134.73, 130.28, 130.09, 128.26, 106.19, 52.26, 17.23.

*Synthesis of **4***: Compound **3** (454 mg, 2 mmol) was dissolved in a mixed solvent of EtOH-THF (7 mL-10 mL), then sodium borohydride (19 mg, 0.5 mmol) was added

slowly to the reaction mixture at 0 °C. After the reaction is completed, water is added to quench, extracted with ethyl acetate, and concentrated under vacuo. The residue was purified by silica gel flash column chromatography to get a white crystalline solid (412 mg, 90%). ^{1}H NMR (400 MHz, DMSO-d_6) δ 7.62 (d, J = 6.8 Hz, 1H), 7.37 – 7.15 (m, 5H), 6.13 (t, J = 6.7 Hz, 1H), 5.22 (s, 1H), 5.09 (s, 2H), 4.49 (s, 2H), 2.01 (s, 3H). ^{13}C NMR (101 MHz, DMSO-d_6) δ 161.94, 141.89, 136.93, 136.25, 135.93, 128.46, 127.67, 126.67, 105.10, 62.74, 51.20, 17.06.

Synthesis of **5:** Compound **4** (458 mg, 2 mmol) was dissolved in CH_2Cl_2 (50 mL), and phosphorus tribromide (1.08 g, 4 mmol) was added to the solution. The reaction mixture was stirred at room temperature overnight. After the reaction is completed, the mixture was diluted with chloroform (100 mL), and wash with saturated aqueous $NaHCO_3$ (30 mL), water (30 mL) and brine (30 mL). The organic layer was dried over anhydrous sodium sulfate and concentrated under reduced pressure. The residue was purified by silica gel flash column chromatography to obtain white solids (465 mg, 80%).^{1}H NMR (400 MHz, Chloroform-d) δ 7.23 (d, J = 8.2 Hz, 2H), 7.16 (d, J = 8.1 Hz, 2H), 7.08 (d, J = 6.8 Hz, 2H), 5.97 (t, J = 6.8 Hz, 1H), 5.00 (s, 2H), 4.34 (s, 2H), 2.05 (s, 3H). ^{13}C NMR (101 MHz, $CDCl_3$) δ 162.77, 137.24, 136.89, 136.67, 134.63, 129.99, 129.35, 128.34, 105.81, 51.87, 33.00, 17.26.

Synthesis of ***6:*** Compound **5** (580 mg, 2 mmol) and PPh_3 (629 mg, 2.4 mmol) were dissolved in toluene (10 mL) and heated to reflux for 18h. Upon completion of the reaction, the mixture was allowed to cool to room temperature, and the resulting white solid was collected by filtration. The solid was washed with toluene, then dried under

vacuum distillation got the final product, a white crystalline solid (443 mg, 40%).^{1}H NMR (400 MHz, Chloroform-*d*) δ 7.65 – 7.43 (m, 15H), 7.30 (s, 1H), 7.07 (s, 1H), 6.90 (s, 4H), 5.98 (t, *J* = 6.8 Hz, 1H), 5.08 (d, *J* = 13.3 Hz, 4H), 4.98 (s, 2H), 1.90 (s, 3H). ^{13}C NMR (101 MHz, $CDCl_3$) δ 161.80, 136.43, 136.18, 134.65, 134.16, 134.13, 133.35, 133.25, 130.68, 130.63, 129.29, 129.17, 128.37, 127.17, 127.14, 125.65, 125.57, 116.89, 116.04, 105.11, 50.88, 29.34, 28.87, 16.36.

*Synthesis of **Endo-py-tpp-1:*** In an oxygen atmosphere of 0 °C, 277 mg of **6** was dissolved in 2 mL of dichloromethane, and a catalytic amount of methylene blue was added. During the reaction, red light irradiation (18 W, 625 nm) was used. After the reaction was completed, the solvent was distilled under low temperature and purified by column chromatography to afford a white solid (278 mg, 95%). ^{1}H NMR (400 MHz, Chloroform-*d*) δ 7.84 – 7.53 (m, 15H), 7.09 – 6.94 (m, 4H), 6.77 (m, 1H), 6.43 (dd, *J* = 7.8, 1.9 Hz, 1H), 5.60 (dd, *J* = 5.3, 1.9 Hz, 1H), 5.42 – 5.26 (m, 2H), 4.67 (s, 1H), 4.45 (s 1H), 1.61 (s, 3H).

*Synthesis of **8:*** 4-bromomethylphenylacetic acid (1.14 g, 5 mmol) was dissolved in THF (20 mL) and slowly added to 500 uL of a 10 M solution of borane (BH) in THF under stirring at 0°C. The reaction mixture was then gradually warmed to room temperature and stirred overnight. After the reaction was completed, 20 mL water was added to the mixture very slowly, and extracted with ethyl acetate. The organic phase was dried over anhydrous magnesium sulfate, filtered, and concentrated under reduced pressure to afford a white crystalline solid (750 mg, 70%). ^{1}H NMR (400 MHz, Chloroform-*d*) δ

7.23 (d, *J* = 8.0 Hz, 2H), 7.09 (d, *J* = 7.9 Hz, 2H), 4.38 (s, 2H), 3.69 (t, *J* = 6.6 Hz, 2H), 2.73 (t, *J* = 6.6 Hz, 2H), 2.20 (s, 1H). ^{13}C NMR (101 MHz, $CDCl_3$) δ 139.10, 135.90, 129.48, 129.23, 63.34, 38.80, 33.59.

Synthesis of ***9:*** K_2CO_3 (0.7 g, 2.07 mmol), compound **8** (428 mg, 2 mmol) and 3-methyl-2-pyridone (182 mg, 1.67 mmol) dissolved in acetonitrile. After adding catalytic amount of 18-benzocrown-6 and KI, reaction was left to stir and reflux. The progress of the reaction was monitored by TLC. When the reaction was completed, concentrated under vacuo. The residue was purified by silica gel flash column chromatography to get a white powder (301 mg, 62%). ^{1}H NMR (400 MHz, Chloroform-*d*) δ 7.14 – 7.01 (m, 6H), 5.98 (t, *J* = 6.7 Hz, 1H), 4.96 (s, 2H), 4.01 – 3.71 (s, 1H), 3.64 (t, *J* = 7.0 Hz, 2H), 2.69 (t, *J* = 7.0 Hz, 2H), 2.04 (s, 3H). ^{13}C NMR (101 MHz, $CDCl_3$) δ 162.75, 138.60, 136.82, 134.62, 134.00, 129.47, 129.10, 127.83, 105.95, 62.88, 51.82, 38.56, 17.13.

Synthesis of ***10:*** Compound **9** (486 mg, 2 mmol) and CBr_4 (730 mg, 2.2 mmol) were dissolved in DCM (10mL), then triphenylphosphine (576 g, 22 mmol) was added slowly to the reaction at 0 °C. The reaction mixture is warmed to room temperature and stirred overnight. Upon completion, the mixture was diluted with chloroform (100 mL) and wash with brine (30 mL). The organic layer was dried over anhydrous sodium sulfate and concentrated under reduced pressure. The residue was purified by silica gel flash column chromatography to get a light yellow oil (500mg, 70%). ^{1}H NMR (400 MHz, Chloroform-*d*) δ 7.17 (d, *J* = 7.9 Hz, 2H), 7.13 – 7.03 (m, 4H), 5.96 (t, *J* = 6.8 Hz, 1H), 5.00 (s, 2H), 3.41 (t, *J* = 7.5 Hz, 2H), 3.00 (t, *J* = 7.5 Hz, 2H), 2.06 (s, 3H).

^{13}C NMR (101 MHz, Chloroform-*d*) δ 162.58, 138.16, 136.46, 135.06, 134.53, 129.63, 128.75, 128.05, 105.56, 51.70, 38.58, 32.60, 17.09.

*Synthesis of **11:*** Compound **10** (610 mg, 2 mmol) and PPh_3 (629 mg, 2.4 mmol) were dissolved in toluene (10 mL) and heated to reflux for 4 days. The mixture was allowed to cool to room temperature and solvent removed off. Hexane (30.0 mL) was added with stirring at 0 °C. The mixture was stirred for 30 mins before removing hexane off and scratching at the gum with a spatula to induce mobility. This process was repeated 5 times. After removing hexane, the mixture was dried under reduced pressure at 45 °C, product was obtained as white solid (460 mg, 40%). ^1H NMR (400 MHz, Chloroform-*d*) δ 7.74 (m, 15H), 7.30 – 7.12 (m, 6H), 6.10 (s, 1H), 5.03 (s, 2H), 4.03 (s, 2H), 3.05 (s, 2H), 2.08 (s, 3H). ^{13}C NMR (101 MHz, $CDCl_3$) δ 162.22, 137.10, 136.34, 134.97, 134.60, 133.10, 133.00, 130.02, 129.89, 128.38, 128.00, 124.66, 117.67, 116.82, 105.39, 51.20, 27.36, 24.51, 23.62, 16.74.

*Synthesis of **Endo-py-tpp-2:*** The method is the same as the synthesis of compound **Endo-py-tpp-1**. White solid, yield 97%. ^1H NMR (400 MHz, Chloroform-*d*) δ 7.82 – 7.71 (m, 9H), 7.64 – 7.60 (m, 6H), 7.26 – 7.19 (m, 2H), 7.07 – 6.96 (d, *J* = 7.8 Hz, 2H), 6.72 (m, 1H), 6.35 (dd, *J* = 7.8, 1.9 Hz, 1H), 5.51 (dd, *J* = 5.3, 1.9 Hz, 1H), 4.65 (d, *J* = 15.3 Hz, 1H), 4.29 (d, *J* = 15.4 Hz, 1H), 4.07 – 3.86 (m, 2H), 2.94 (m, 2H), 1.99 (s, 3H).

*Synthesis of **13:*** Compound **12** (330 mg, 2 mmol) was dissolved in CH_3CN, then NBS (531 mg, 2.4 mmol) and AIBN (33 mg, 0.2 mmol) were added and heated to reflux. When the reaction completed, cooled to room temperature, the solvent was removed under reduced pressure. Then the solid mixture was dispersed in a combination of water

and ethyl acetate. The organic phase was subsequently collected, dried over anhydrous sodium sulfate, and the solvent was removed under reduced pressure. The residue was purified by silica gel flash column chromatography to get a light yellow oil (400 mg, 83%). ^{1}H NMR (400 MHz, Chloroform-*d*) δ 7.33 (d, *J* = 7.9 Hz, 2H), 7.19 (d, *J* = 7.9 Hz, 2H), 4.49 (s, 2H), 2.96 (t, *J* = 7.7 Hz, 2H), 2.69 (t, *J* = 7.7 Hz, 2H). ^{13}C NMR (101 MHz, CDC13) δ ^{13}C NMR (101 MHz, $CDCl_3$) δ 179.17, 140.65, 136.04, 129.41, 128.87, 35.48, 33.53, 30.33.

*Synthesis of **14:*** The method is the same as the synthesis of compound **8**. White powder, yield 70%. ^{1}H NMR (400 MHz, Chloroform-*d*) δ 7.22 (d, *J* = 7.9 Hz, 2H), 7.08 (d, *J* = 8.0 Hz, 2H), 4.40 (s, 2H), 3.57 (t, *J* = 6.4 Hz, 2H), 2.61 (t, *J* = 7.4 Hz, 2H), 1.89 (s, 1H), 1.84 – 1.74 (m, 2H). ^{13}C NMR (101 MHz, $CDCl_3$) δ 142.38, 135.39, 129.19, 128.94, 62.12, 34.06, 33.76, 31.83.

*Synthesis of **15:*** The method is the same as the synthesis of compound **9**. White powder, yield 65%. ^{1}H NMR (400 MHz, Chloroform-*d*) δ 7.08 – 6.94 (m, 6H), 5.94 (t, *J* = 6.7 Hz, 1H), 4.94 (s, 2H), 3.47 (t, *J* = 6.5 Hz, 2H), 2.51 (t, *J* = 7.9 Hz, 2H), 2.01 (s, 3H), 1.69 (m, 2H). ^{13}C NMR (101 MHz, $CDCl_3$) δ 162.79, 141.73, 136.79, 134.56, 133.55, 129.49, 128.58, 127.88, 105.95, 61.29, 51.77, 33.89, 31.51, 17.09.

*Synthesis of **16:*** The method is the same as the synthesis of compound **10**. light yellow oil, yield 78%. ^{1}H NMR (400 MHz, Chloroform-*d*) δ 7.20 – 6.99 (m, 6H), 5.98 (t, *J* = 6.8 Hz, 1H), 5.02 (s, 2H), 3.26 (t, *J* = 6.6 Hz, 2H), 2.65 (t, *J* = 7.4 Hz, 2H), 2.13 – 1.95 (m, 5H). ^{13}C NMR (101 MHz, $CDCl_3$) δ 162.66, 139.93, 136.45, 134.53, 134.34, 129.69, 128.69, 128.09, 105.56, 51.75, 33.74, 33.31, 32.84, 17.14.

Synthesis of ***17:*** The method is the same as the synthesis of compound **11**. White solid, yield 45% ^{1}H NMR (400 MHz, Chloroform-*d*) δ 7.72 – 7.46 (m, 15H), 7.30 (d, *J* = 6.8 Hz, 1H), 7.15 – 6.94 (m, 5H), 5.98 (t, *J* = 6.8 Hz, 1H), 4.99 (s, 2H), 3.62 (t, *J* = 16.4 Hz, 1H), 2.83 (t, *J* = 7.2 Hz, 2H), 1.95 (s, 3H), 1.87 – 1.72 (m, 3H). ^{13}C NMR (101 MHz, $CDCl_3$) δ 161.76, 138.49, 135.94, 134.43, 134.08, 134.05, 133.95, 132.49, 132.40, 129.53, 129.41, 128.29, 128.08, 127.30, 117.28, 116.42, 104.92, 50.91, 34.27, 34.10, 23.45, 20.65, 20.15, 16.27.

Synthesis of ***Endo-py-tpp-3:*** The method is the same as the synthesis of compound. **Endo-py-tpp-1**. Light yellow solid, yield 96%. ^{1}H NMR (400 MHz, Chloroform-*d*) δ 7.80 – 7.60 (m, 15H), 7.20 – 7.04 (m, 4H), 6.75 (m, 1H), 6.42 (dd, *J* = 7.8, 1.8 Hz, 1H), 5.59 (dd, *J* = 5.3, 1.8 Hz, 1H), 4.69 (d, *J* = 15.3 Hz, 1H), 4.45 (d, *J* = 15.3 Hz, 1H), 3.84 – 3.66 (m, 1H), 2.98 (t, *J* = 7.2 Hz, 2H), 1.97-1.84 (m, 5H).

Synthesis of ***19:*** The method is the same as the synthesis of compound **13**.

Synthesis of ***20:*** The method is the same as the synthesis of compound **8**. Light yellow oil, yield 70%. ^{1}H NMR (400 MHz, Chloroform-*d*) δ 7.20 (d, *J* = 7.8 Hz, 2H), 7.05 (d, *J* = 7.8 Hz, 2H), 4.38 (s, 2H), 3.54 (t, *J* = 6.3 Hz, 2H), 3.39 – 3.36 (s, 1H), 2.53 (t, *J* = 7.4 Hz, 2H), 1.63 – 1.45 (m, 4H). ^{13}C NMR (101 MHz, $CDCl_3$) δ 142.79, 135.19, 129.05, 128.84, 62.55, 35.30, 33.79, 32.06, 27.39.

Synthesis of ***21:*** The method is the same as the synthesis of compound **9**.

Synthesis of ***22:*** The method is the same as the synthesis of compound **10**. White powder, yield 60%. ^{1}H NMR (400 MHz, Chloroform-*d*) δ 7.15 – 6.95 (m, 6H), 5.92 (t, *J* = 6.8 Hz, 1H), 4.97 (s, 2H), 3.26 (t, *J* = 6.7 Hz, 2H), 2.47 (t, *J* = 7.6 Hz, 2H), 2.03 (s,

3H), 1.73 (m, 2H), 1.61 (m, 2H). ^{13}C NMR (101 MHz, $CDCl_3$) δ 162.65, 141.20, 136.37, 134.50, 134.04, 129.65, 128.51, 128.02, 105.48, 51.70, 34.30, 33.42, 31.88, 29.45, 17.14.

Synthesis of ***23:*** The method is the same as the synthesis of compound **11**. ^{1}H NMR (400 MHz, Chloroform-*d*) δ 7.76 – 7.49 (m, 15H), 7.24 – 6.91 (m, 6H), 6.02 (t, J = 6.8 Hz, 1H), 4.99 (s, 2H), 3.58 (t, J = 9.2 Hz ,2H), 2.52 (t, J = 7.5 Hz, 2H), 2.04 (s, 3H), 1.85 (t, J = 7.7 Hz, 2H), 1.58 – 1.50 (m, 2H). ^{13}C NMR (101 MHz, $CDCl_3$) δ 162.61, 140.43, 136.54, 134.87, 134.79, 134.76, 134.71, 133.28, 133.18, 130.26, 130.14, 128.53, 127.91, 118.16, 117.30, 105.70, 51.53, 33.97, 30.93, 30.77, 22.33, 21.83, 21.30, 21.26, 17.05.

Synthesis of ***Endo-py-tpp-4:*** The method is the same as the synthesis of compound **Endo-py-tpp-1**. Light yellow solid, yield 96%. ^{1}H NMR (400 MHz, Chloroform-*d*) δ 7.80 – 7.58 (m, 15H), 7.06 – 6.96 (m, 4H), 6.67 (m, 1H), 6.39 (m, 1H), 5.43 (m, 1H), 4.73 (d, J = 15.3 Hz, 1H), 4.29 (d, J = 15.3 Hz, 1H), 3.83 (m, 3H), 2.58 (t, J = 7.5 Hz, 2H), 1.93 (m, 1H), 1.59 (m, 5H).

3. Temporal Evolution of endoperoxides ^{1}H NMR

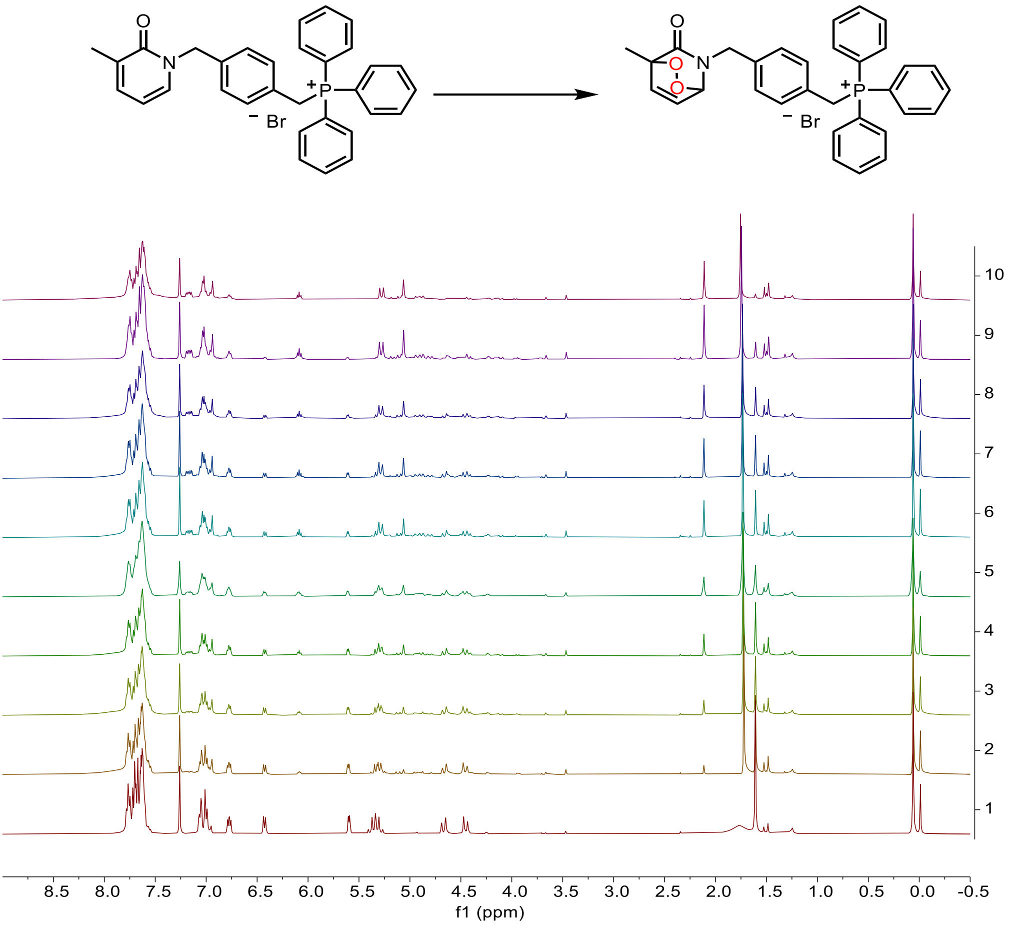

Fig S1. Temporal evolution of **Endo-py-tpp-1** ^{1}H NMR in $CDCl_3$ at 37 °C. (Spectra 1 - 0 hours, Spectra 2 - 2 hours, Spectra 3 - 4 hours, Spectra 4 - 6 hours, Spectra 5 - 8 hours, Spectra 6 - 10 hours, Spectra 7 - 12 hours, Spectra 8 - 14 hours, Spectra 9 - 24 hours, Spectra 10- 36 hours).

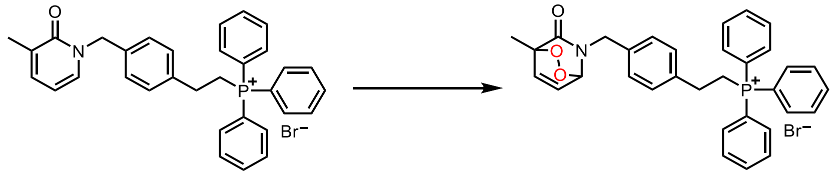

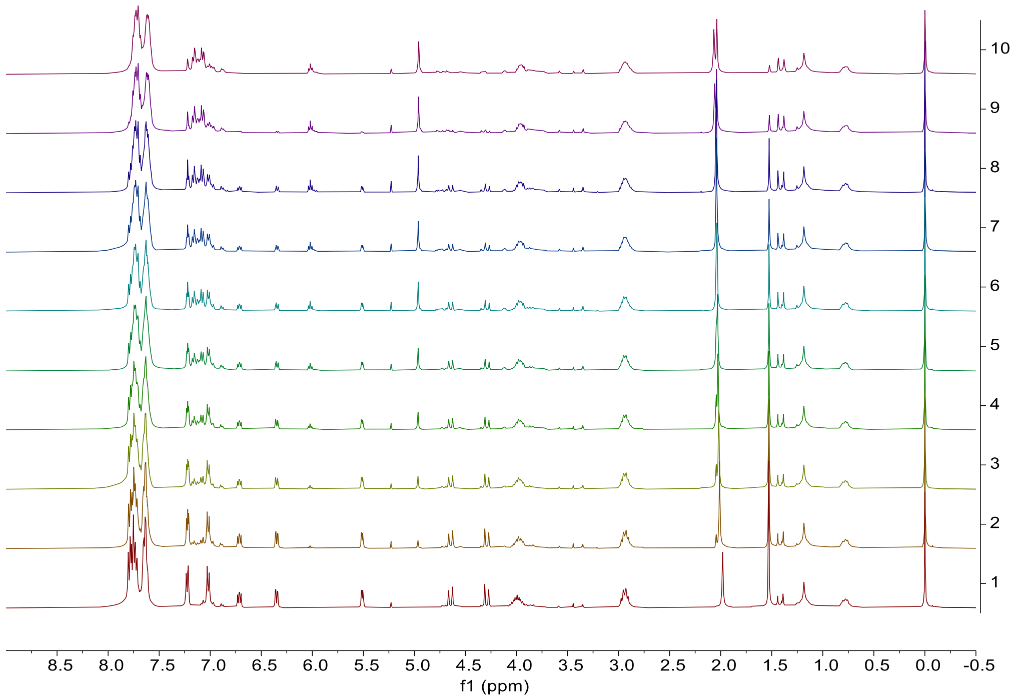

Fig S2. Temporal evolution of **Endo-py-tpp-2** ^{1}H NMR in $CDCl_3$ at 37 °C. (Spectra 1 - 0 hours, Spectra 2 - 2 hours, Spectra 3 - 4 hours, Spectra 4 - 6 hours, Spectra 5 - 8 hours, Spectra 6 - 10 hours, Spectra 7 - 12 hours, Spectra 8 - 14 hours, Spectra 9 - 24 hours, Spectra 9- 30 hours).

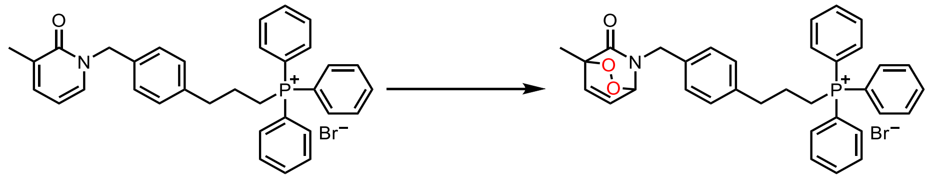

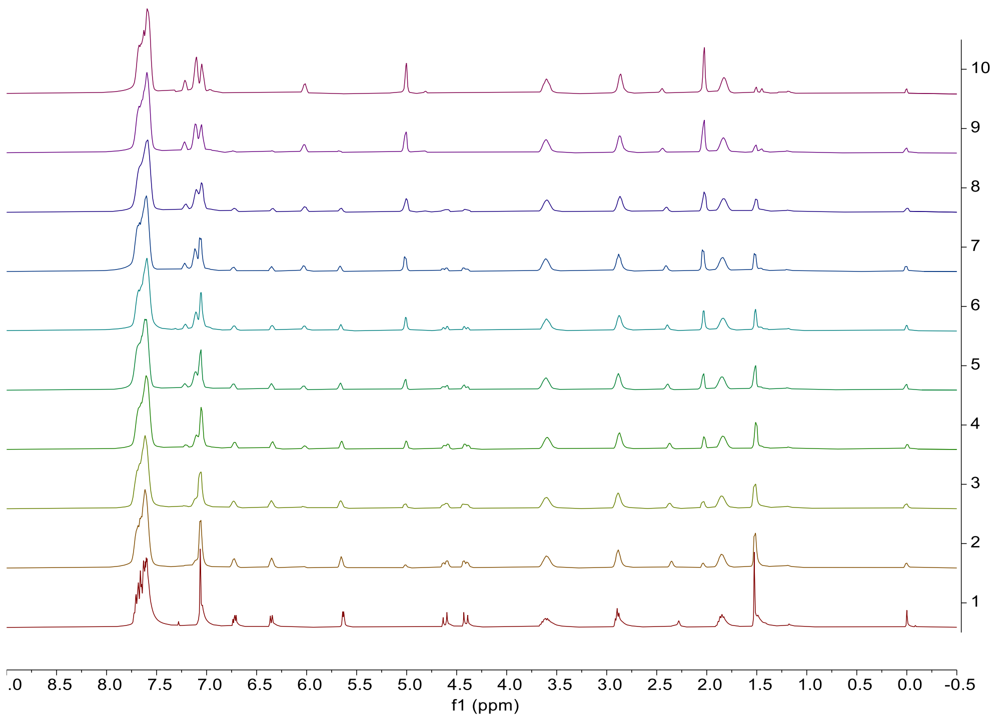

Fig S3. Temporal evolution of **Endo-py-tpp-3** ^{1}H NMR in $CDCl_3$ at 37 °C. (Spectra 1 - 0 hours, Spectra 2 - 2 hours, Spectra 3 - 4 hours, Spectra 4 - 6 hours, Spectra 5 - 8 hours, Spectra 6 - 10 hours, Spectra 7 - 12 hours, Spectra 8 - 14 hours, Spectra 9 - 24 hours, Spectra 9- 30 hours).

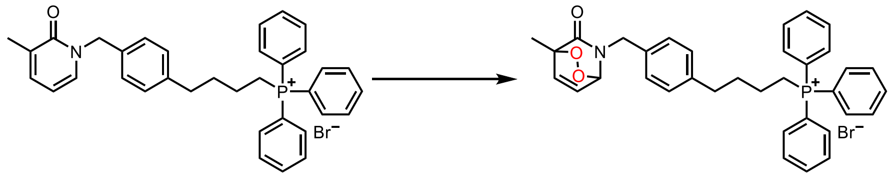

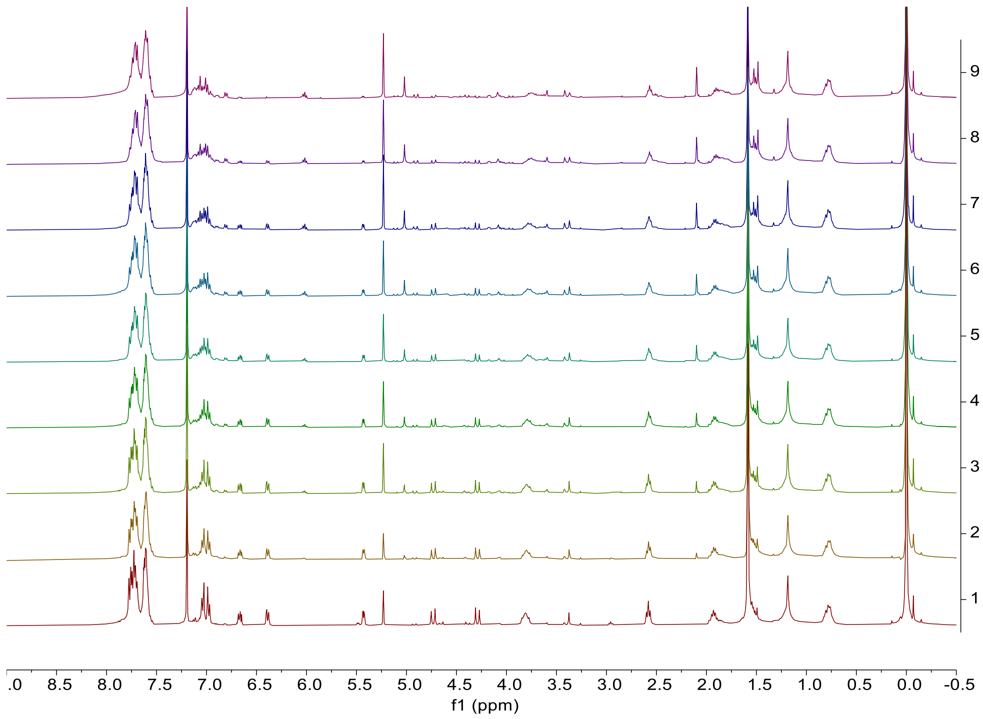

Fig S4. Temporal evolution of **Endo-py-tpp-4** ^{1}H NMR in $CDCl_3$ at 37 °C. (Spectra 1 - 0 hours, Spectra 2 - 1 hours, Spectra 3 - 2 hours, Spectra 4 - 3 hours, Spectra 5 - 4 hours, Spectra 6 - 5 hours, Spectra 7 - 6 hours, Spectra 8 - 8 hours, Spectra 9 - 10 hours).

The half-life of compound **endoperoxides** was calculated from the first-order reaction rate equation in ^{1}H NMR. The calculation formula is as follows: $\ln[A] = -kt + \ln[A]_0$, $t_{1/2} = 0.693/k$

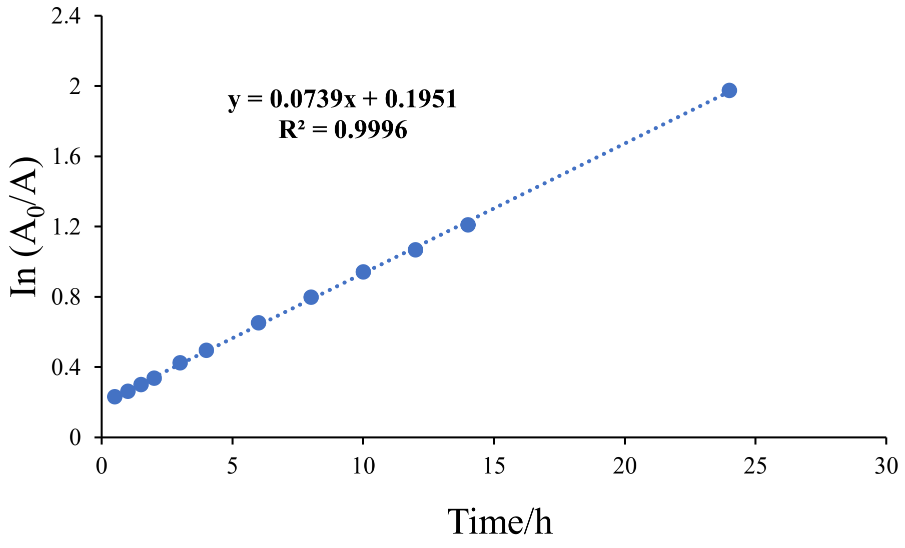

Fig S5. Half-life calculation of **Endo-py-tpp-1**: 9.4 hours (at 37 °C in $CDCl_3$)

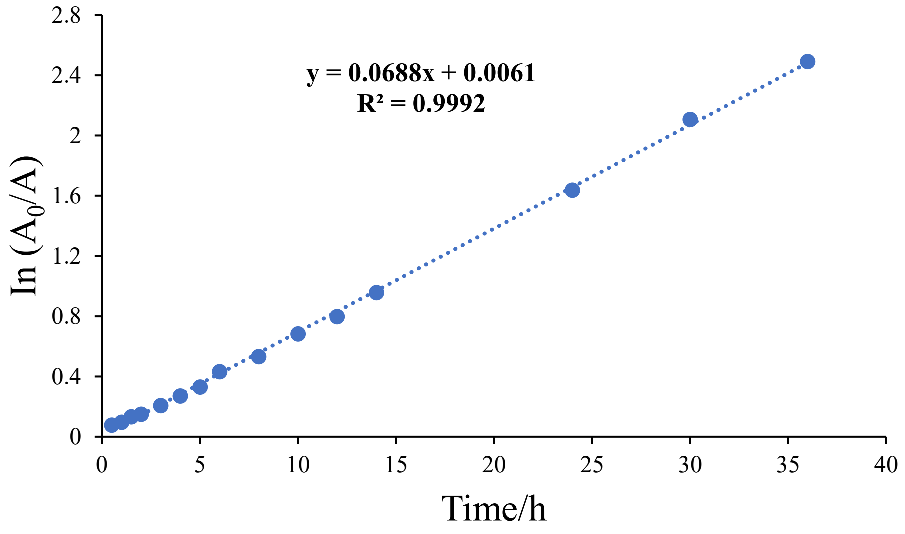

Fig S6. Half-life calculation of **Endo-py-tpp-2**: 10.07 hours (at 37 °C in $CDCl_3$)

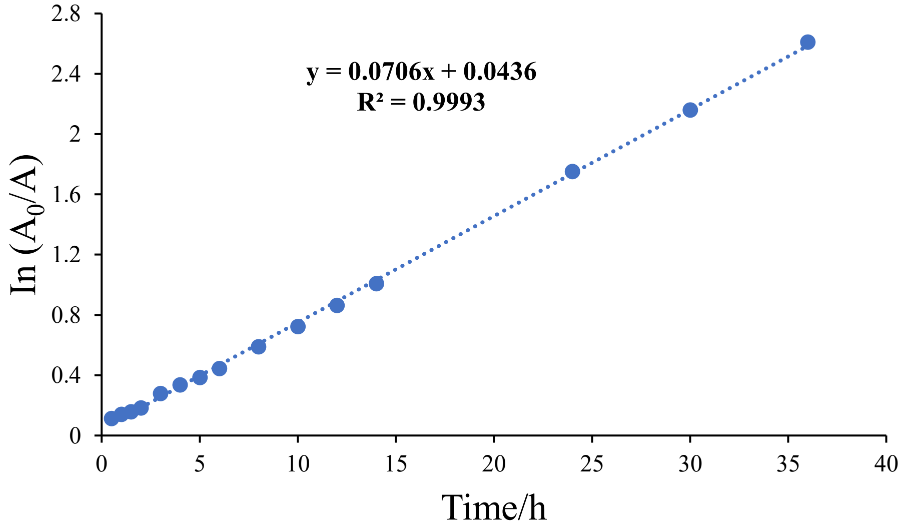

Fig S7. Half-life calculation of **Endo-py-tpp-3**: 9.8 hours (at 37 °C in $CDCl_3$)

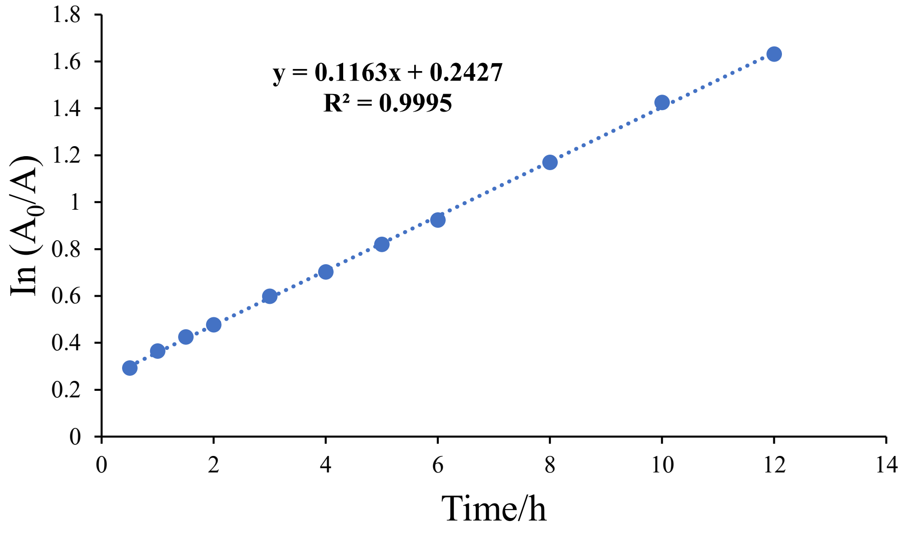

Fig S8. Half-life calculation of **Endo-py-tpp-4**: 5.9 hours (at 37 °C in $CDCl_3$)

4. Detection of singlet oxygen release

To detect singlet oxygen release of endoperoxides, 1,3-Diphenylisobenzofuran was used as a singlet oxygen probe. In a 4 ml cuvette, DMF were used as the test solvent, then DPBF (50 μM, final concentration), **endoperoxides** (1 mM, final concentration) was added and stirred, the absorbance was monitored over time using a UV-Vis absorbance meter. DPBF alone, DPBF with **endoperoxides** were tested. Value of k for the DPBF consuming rate was acquired from the absorption change at 415 nm, and the first-order reaction kinetic formula is:

$$\ln[A] = -kt + \ln[A]_0$$

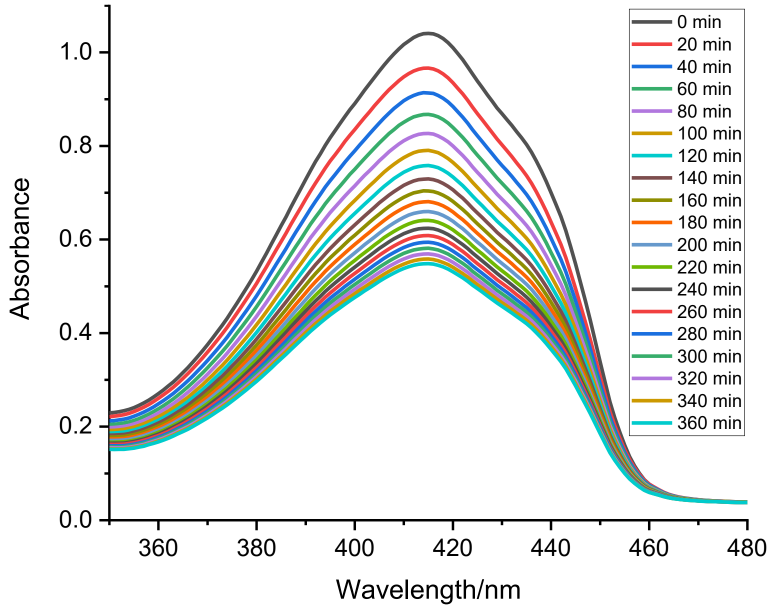

Fig S9. Time dependent UV-Vis spectra of DPBF in the presence of **Endo-py-tpp-1** in DMF at 37 °C.

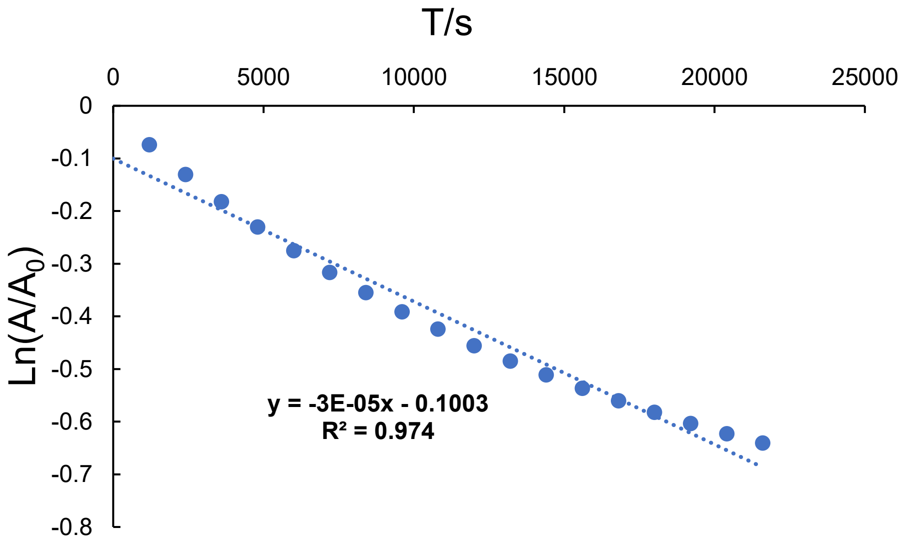

Fig S10. Calculation of the DPBF consumption rate: $k = 3 \times 10^{-5}$ s^{-1}

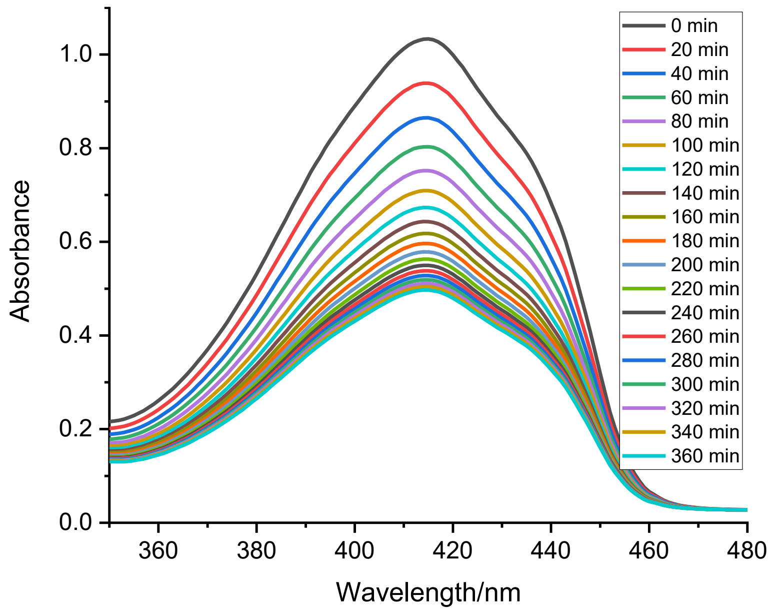

Fig S11. Time dependent UV-Vis spectra of DPBF in the presence of **Endo-py-tpp-2** in DMF at 37 °C.

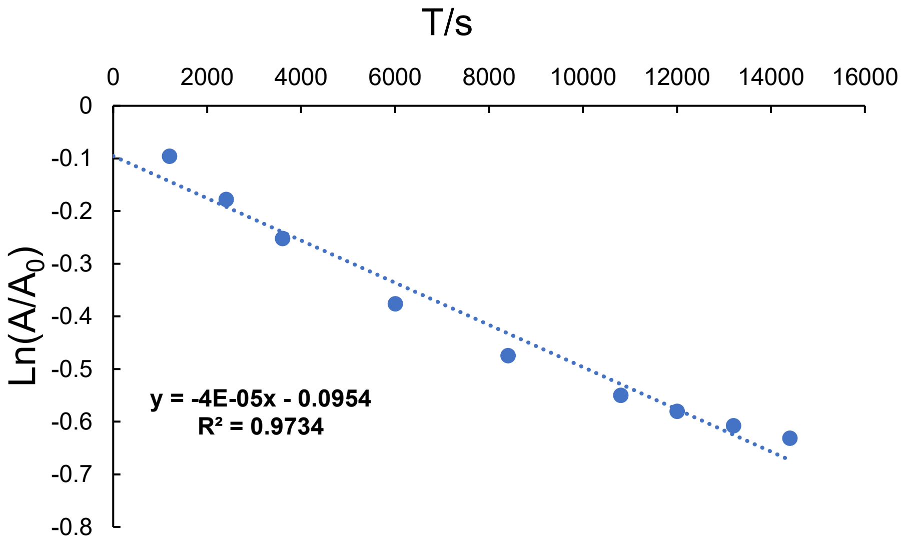

Fig S12. Calculation of the DPBF consumption rate: $k = 4 \times 10^{-5}$ s^{-1}

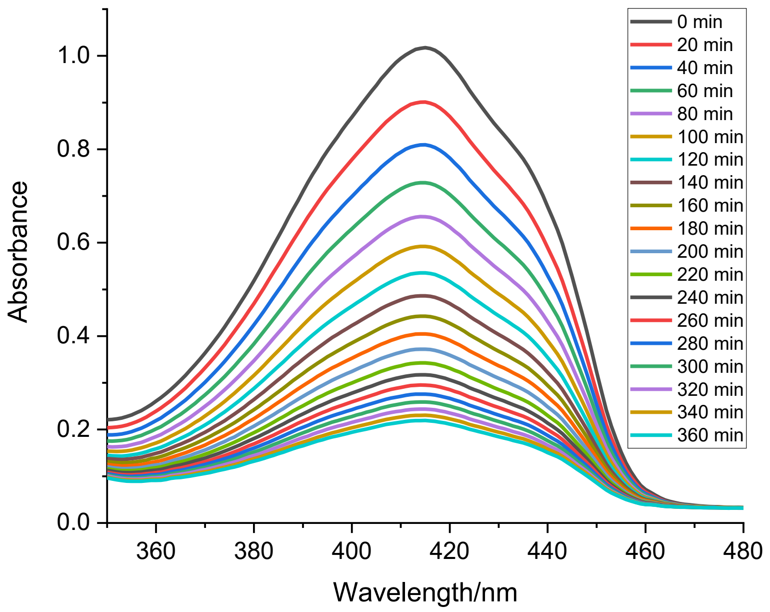

Fig S13. Time dependent UV-Vis spectra of DPBF in the presence of **Endo-py-tpp-3** in DMF at 37 °C.

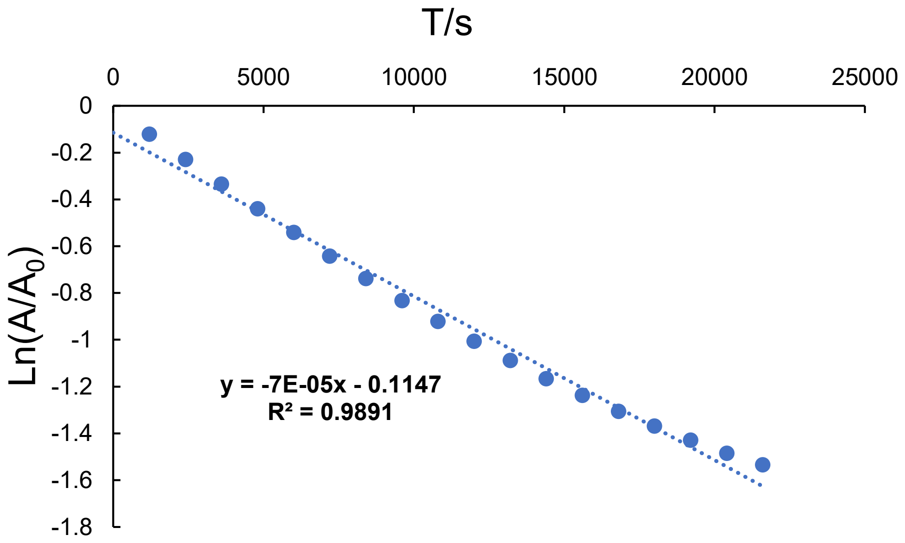

Fig S14. Calculation of the DPBF consumption rate: $k = 7 \times 10^{-5}$ s^{-1}

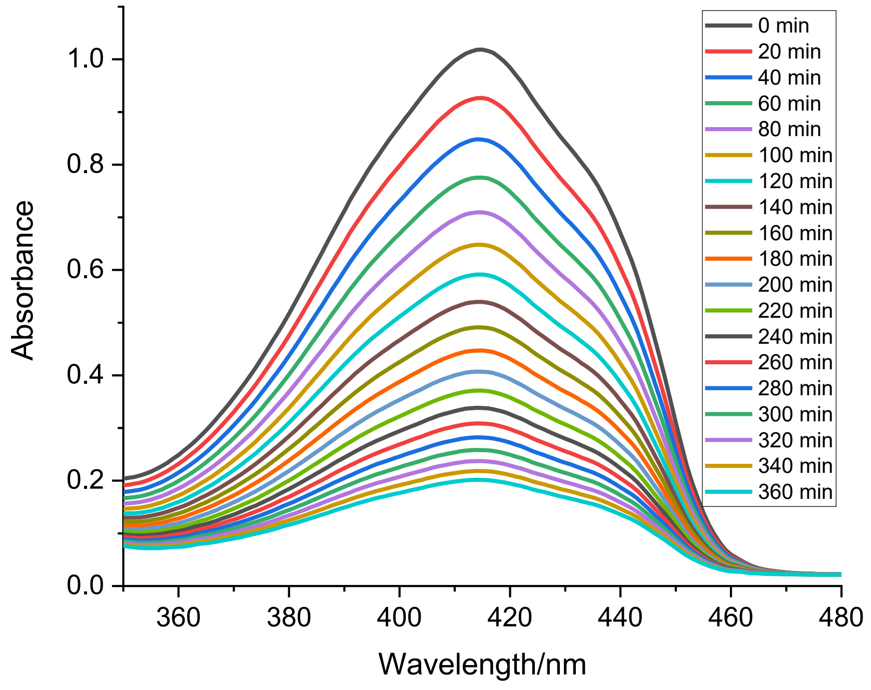

Fig S15. Time dependent UV-Vis spectra of DPBF in the presence of **Endo-py-tpp-4** in DMF at 37 °C.

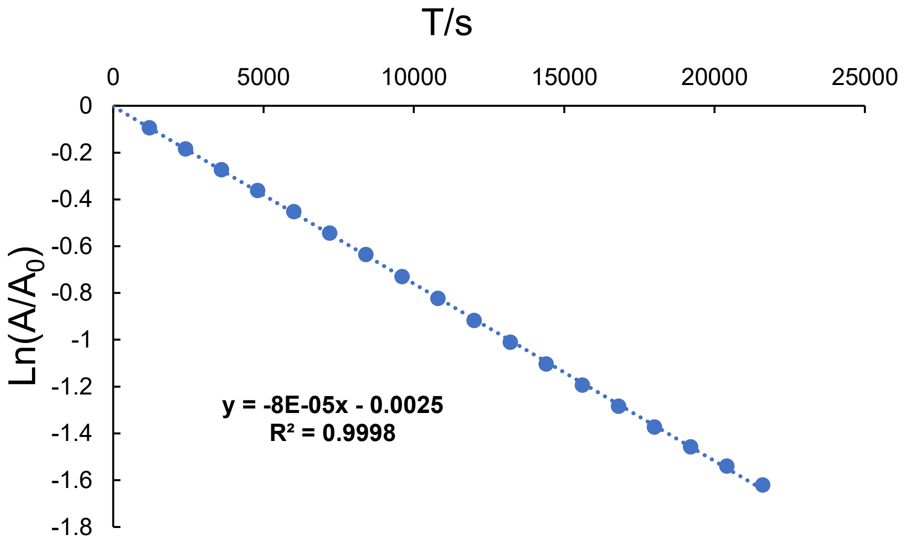

Fig S16. Calculation of the DPBF consumption rate: $k = 8 \times 10^{-5}$ s^{-1}

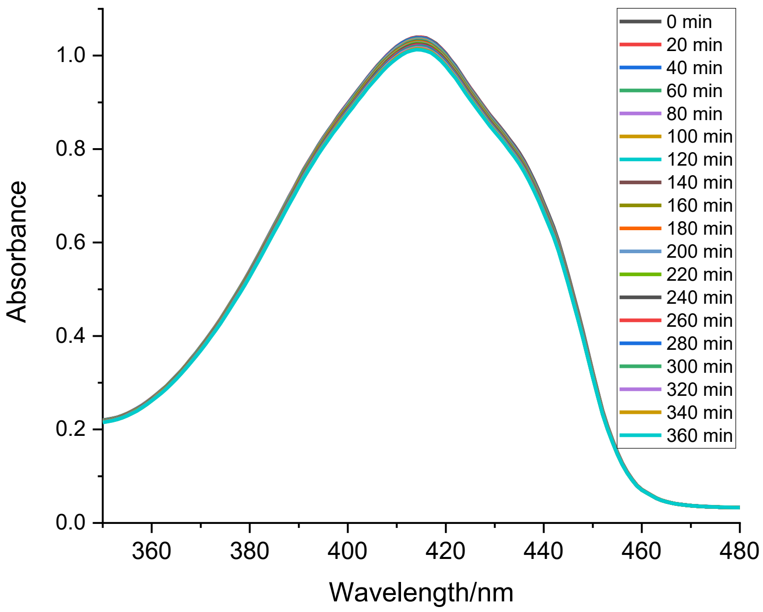

Fig S17. Time dependent UV-Vis spectra of DPBF in DMF at 37 °C.

The singlet oxygen release of **endoperoxides** in PBS was measured using SOSG as a fluorescent probe. In a 4 ml cuvette, PBS were used as the test solvent, then SOSG (2.5 µM, final concentration), **endoperoxides** (50 µM, final concentration) was added, the fluorescence intensity was monitored over time using Eclipse Fluorescence Spectrophotometer. SOSG alone, SOSG with **endoperoxides** were tested.

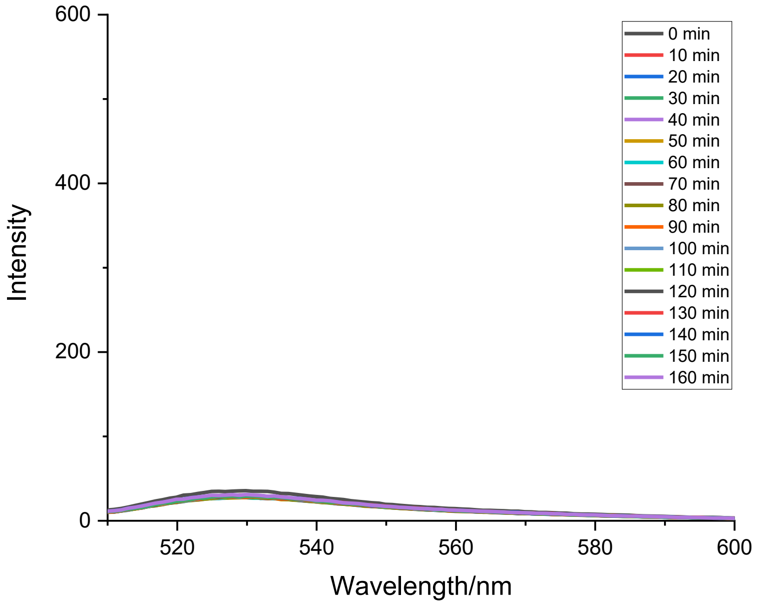

Fig S18. Emission spectrum changes of singlet oxygen probe SOSG (2.5 μM) in PBS.

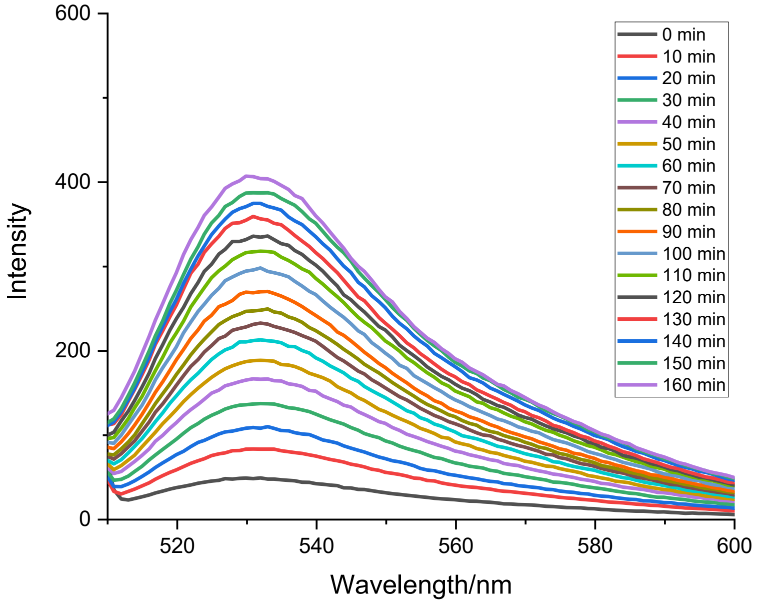

Fig S19. Emission spectrum changes of **Endo-py-tpp-1** (50 μM) in the presence of singlet oxygen probe SOSG (2.5 μM) in PBS.

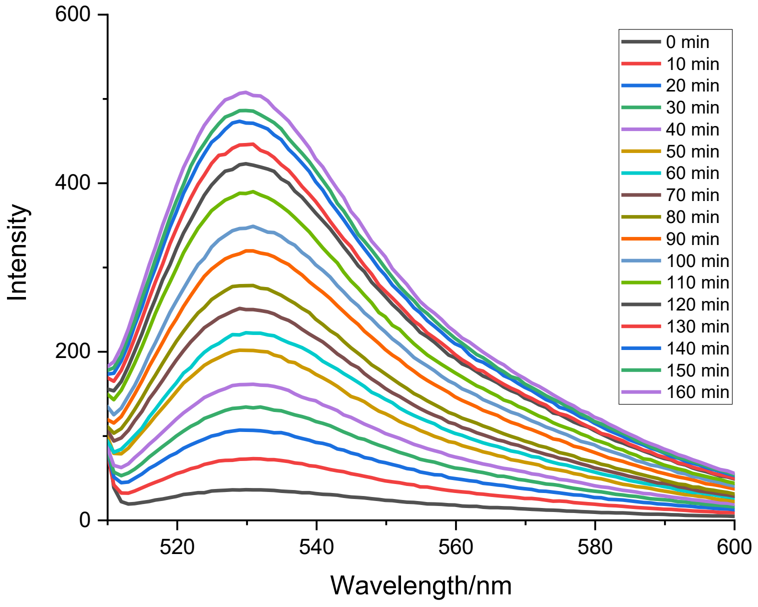

Fig S20. Emission spectrum changes of **Endo-py-tpp-2** (50 μM) in the presence of singlet oxygen probe SOSG (2.5 μM) in PBS.

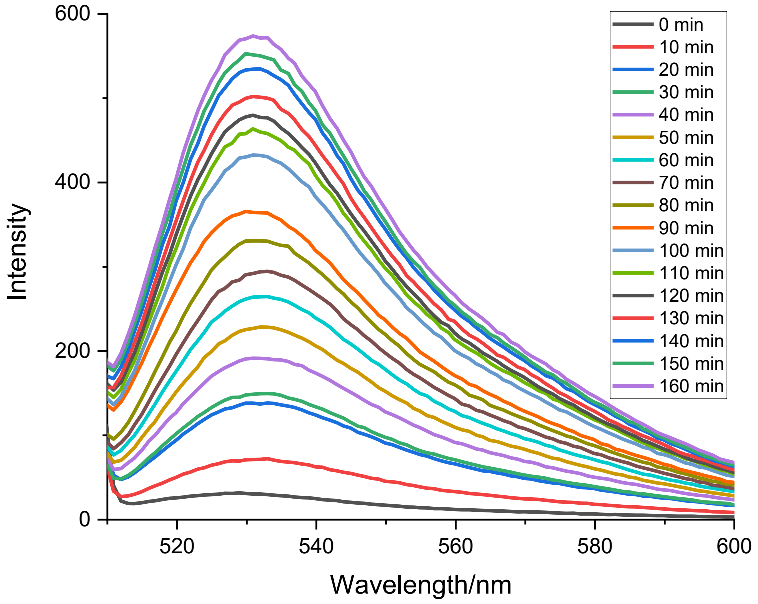

Fig S21. Emission spectrum changes of **Endo-py-tpp-3** (50 μM) in the presence of singlet oxygen probe SOSG (2.5 μM) in PBS.

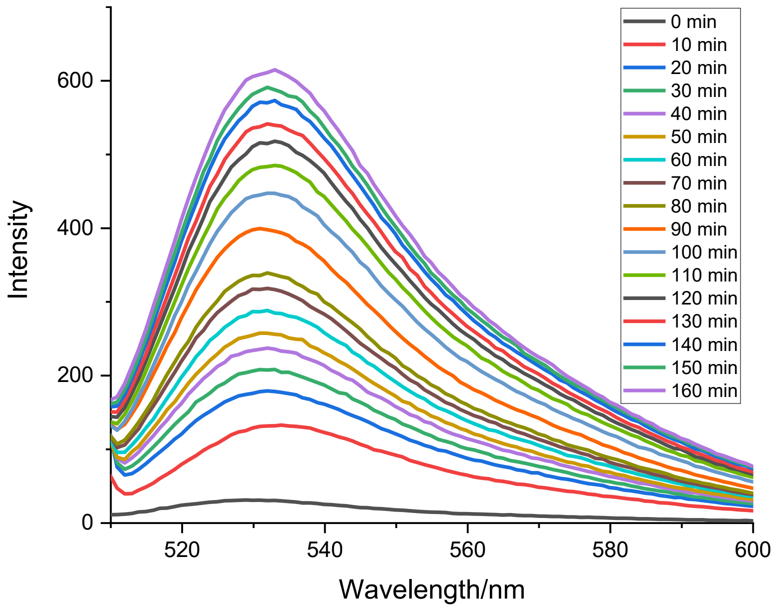

Fig S22. Emission spectrum changes of **Endo-py-tpp-4** (50 μM) in the presence of singlet oxygen probe SOSG (2.5 μM) in PBS.

5. MTT assay

A549, MCF-7, HepG2, HeLa, 4T1 cells, are seeded into 96-well plates at a density of 6000 cells per well. Subsequently, cells were cultured in a carbon dioxide incubator containing 21% oxygen for 24 h. The cells were then treated with different concentrations of endoperoxides for 24 h. It was then incubated with 0.5 mg/mL MTT for 4 h, and finally the medium was removed and 150 ul of DMSO was added to dissolve the formazan crystals. Measure the absorbance at 570 nm with a microplate reader.

6. Detection of singlet oxygen for intracellular generation

In order to assess the intracellular singlet oxygen generation of endoperoxides, we performed fluorescence imaging by using 2',7'-dichlorofluorescein diacetate (DCFH-DA) as reactive oxygen species (ROS) probe. A549 cells (24-well plates at a density of $1.5 * 10^4$ cells per well) were cultured in full growth media at 37 °C with 5 % CO_2 for 24 h in order to achieve proper adhesion of the cells to the glass bottomed dishes. Group 1, incubated with DCFH-DA (10 μM), Hoechst 33342 at 37 °C for 6 h without any treatment (control), group 2, incubated with DCFH-DA (10 μM), Hoechst 33342 and 40 μM **Endo-py-tpp-1** at 37 °C for 6 h; group 3, incubated with DCFH-DA (10 μM), Hoechst 33342 and 40 μM **Endo-py-tpp-2** at 37 °C for 6 h; group 4, incubated with DCFH-DA (10 μM), Hoechst 33342 and 40 μM **Endo-py-tpp-3** at 37 °C for 6 h; group 5, incubated with DCFH-DA (10 μM), Hoechst 33342 and 40 μM **Endo-py-tpp-4** at 37 °C for 6 h;. After incubation, the media were removed and washed with PBS and imaging was observed by High Content Imaging System.

7. Detection of mitochondrial membrane potential

To evaluate the changes in mitochondrial membrane potential, we detected the fluorescence signal changes in A549 cells stained with the JC-1 dye. A549 cells are seeded into 96-well plates at a density of 5,000 cells per well. Subsequently, cells were cultured in a carbon dioxide incubator containing 21% oxygen for 24 h. The cells were then treated with endoperoxides. Group 1, incubated with JC-1 for 20 min without any treatment (control), group 2, incubated with 40 μM **Endo-py-tpp-1** for 6 h, and then incubated with JC-1 for 20 min; group 3, incubated with 40 μM **Endo-py-tpp-2** for 6 h, and then incubated with JC-1 for 20 min; group 4, incubated with 40 μM **Endo-py-tpp-3** for 6 h, and then incubated with JC-1 for 20 min; group 5, incubated with 40 μM **Endo-py-tpp-4** for 6 h, and then incubated with JC-1 for 20 min. After incubation, the media were removed and washed with PBS and imaging was observed by High Content Imaging System.

8. Detection of singlet oxygen within mitochondria

To assay the changes of singlet oxygen within mitochondria, we detected the fluorescence signal changes in A549 cells stained with the Si-DMA. A549 cells are seeded into 96-well plates at a density of 5,000 cells per well. Subsequently, cells were cultured in a carbon dioxide incubator containing 21% oxygen for 24 h. The cells were then treated with endoperoxides. Group 1, incubated with Si-DMA for 40 min without any treatment (control), group 2, incubated with 40 μM **Endo-py-tpp-1** for 6 h, and then incubated with Si-DMA for 40 min; group 3, incubated with 40 μM **Endo-py-tpp-2** for 6 h, and then incubated with Si-DMA for 40 min; group 4, incubated with 40 μM **Endo-py-tpp-3** for 6 h, and then incubated with Si-DMA for 40min; group 5, incubated with 40 μM **Endo-py-tpp-4** for 6 h, and then incubated with Si-DMA for 40 min. After incubation, the media were removed and washed with PBS and imaging was observed by High Content Imaging System.

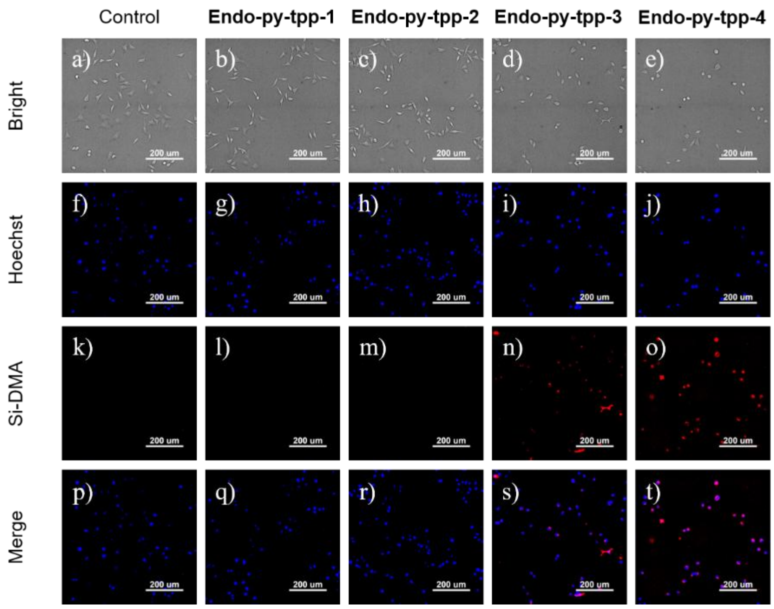

Fig S23. Si-DMA staining with different treatments.

9. Cell apoptosis experiment

To assay the cell apoptosis after endoperoxides treatment, we detected the fluorescence signal changes in A549 cells stained with the Annexin V-FITC. A549 cells are seeded into 6-well plates at a density of $8 * 10^5$ cells per well. Subsequently, cells were cultured in a carbon dioxide incubator containing 21% oxygen for 24 h. The cells were then treated with endoperoxides (40 μM) for 6 h, then A549 cells were digested into a single-cell suspension and resuspended in Annexin V binding buffer. Then Annexin V-FITC and PI were added to stain for 15 min. The data was obtained by flow cytometer

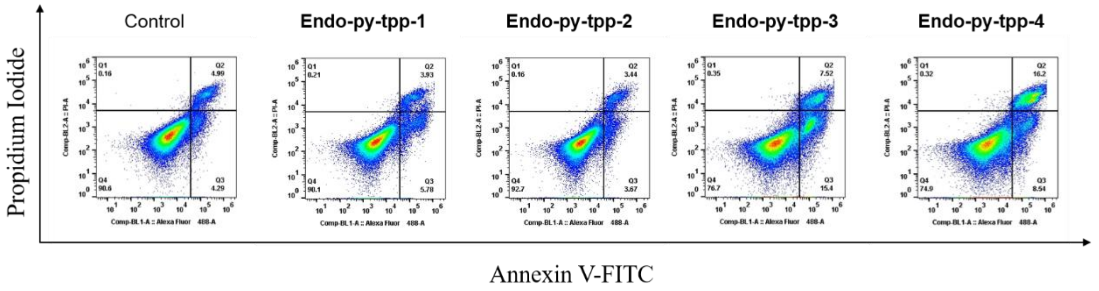

Fig S24. Flow cytometry analysis of apoptosis after different treatments.

10. Live/dead cell staining using Calcein-AM/PI

The live/dead staining assay was performed to visualize the status of A549 cells after being treated by different samples. A549 cells are seeded into 96-well plates at a density of 6000 cells per well. Subsequently, cells were cultured in a carbon dioxide incubator containing 21% oxygen for 24 h. The cells were then treated with endoperoxides (40 μM) for 6 h, washed by PBS. Then Calcein-AM /propidium iodide staining reagents were added and incubated for 20 min. The cells were observed by High Content Imaging System.

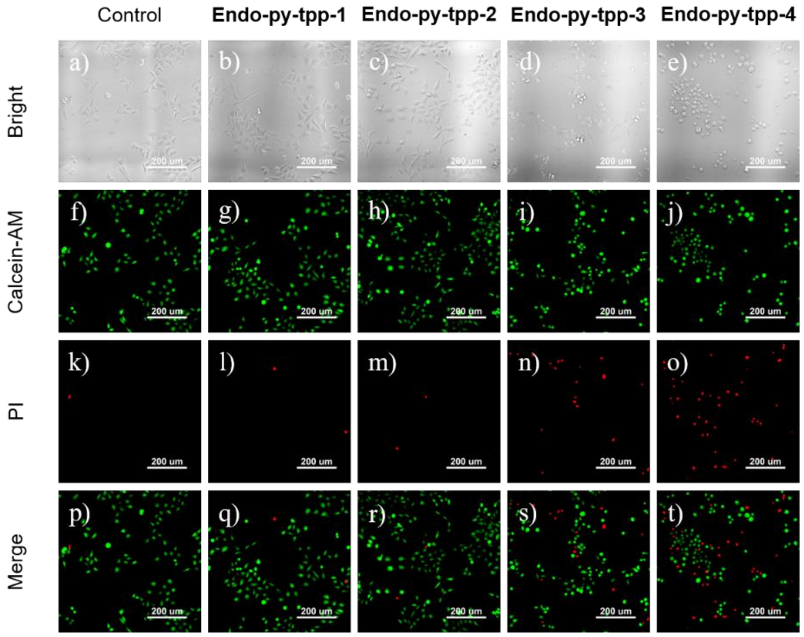

Fig S25. Dead/live staining images with different endoperoxide treatment for 6 hours.

11. 3D (three-dimensional) tumourspheres

A549 cells were used to generate tumor spheroids. Single-cell suspensions (with desired seeding density) were seeded into Nunclon™ Sphera™ 96-well-low attachment plates (Thermo Fisher Scientific) in DMEM-high glucose supplemented with 10% FBS and 1% penicillin/streptomycin. The low attachment plates were incubated for 3 days (37°C, 5% CO_2), treated with endoperoxides (40 μM) for 6h. Then tumor spheroids were washed by PBS, and Calcein-AM/PI were added to stain for 30 min. After incubation, the media were removed and washed with PBS and imaging was observed by High Content Imaging System.

12. Western blot

A549 cells were seeded in 6-well plates (1×10^6 cells/well) and overnight. Cells were pretreated with DMSO and endoperoxides (40 μM) for 24 hours. Then the cells were washed with PBS and lysed by RIPA lysis buffer. Extracted total protein was homogenized in RIPA lysis buffer with protease inhibitor and quantified using a BCA protein assay kit (Shanghai EpiZyme Biotechnology Co., Ltd.). After denaturation, protein samples were transferred to a PVDF membrane after gel electrophoresis and the membrane was blocked with TBST buffer containing 5% non-fat milk. After wash by TBST buffer, PVDF membrane was treated with different primary antibodies: β-actin (1:10000), Caspase-3 (1:1000), Bcl-2 (1:2000) (all from Proteintech, Wuhan, China) at 4 °C overnight. Then, the membranes were incubated with HRP-conjugated secondary antibodies at room temperature for 2 h. After wash with TBST, the protein bands were detected using enhanced chemiluminescent reagents.

13. Colony formation assays

A549 cells were seeded at a density of 1000 cells/well in a six-well plate. The next day, the A549 cells were observed to be fully adherent to the cell wall. Dimethyl sulfoxide (DMSO), endoperoxides (40 μM) were added to the medium. The fluid was changed every 3 d, the cell mass (counting ≥50 cells) was observed under the microscope, the cell mass was visible to the naked eye on the cell culture dish, and cell cloning was performed. Cells in each well were fixed with 4% paraformaldehyde for 20 min, washed three times with PBS, stained with 0.1% crystal violet solution for 15 min, washed again with PBS three times, dried, and photographed.

14. Wound healing scratch assay

A549 cells were seeded in 6-well plates (5×10^5 cells/well) and after 24 h with acceptable confluency indicating a monolayer (~ 80 to 90% confluency), 3 parallel scratches were made on the bottom of the dish using a 10 uL pipette tip. Thereafter, the detached cells were removed with PBS and immediately the cells were treated with the effective concentrations of **endoperoxides** (40 μM) and/or control (medium containing the equal amount of test compounds solvent). Scratch photographs were taken at marked positions after 0 h, 24 h, and 48 h using an inverted fluorescence microscope. The scratch distances were quantified using ImageJ software.

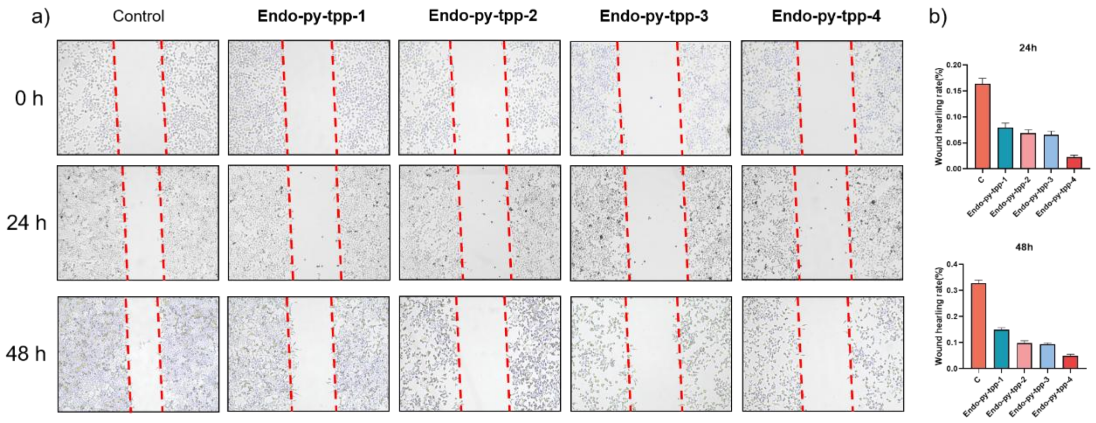

Fig S26. Micrographs of cell migration (a) and wound healing rate (b: 24 h, c: 48 h) were scratched and treated with endoperoxides.

15. Establishment of tumor model

4T1 cells were injected subcutaneously into the armpit of 5–6 week old female BALB/c mice to prepare tumor-bearing mice, the tumor-bearing mice of the models was raised for approximately 8 days, then divided into three groups (five mice in each group): the first group was the control group, the second group was treated with **Endo-py-tpp-1** (14 mg/kg), and the third group was treated with **Endo-py-tpp-4** (15 mg/kg). 15 days later, dissection, removal of tumors and internal organs, and follow-up tests were performed.

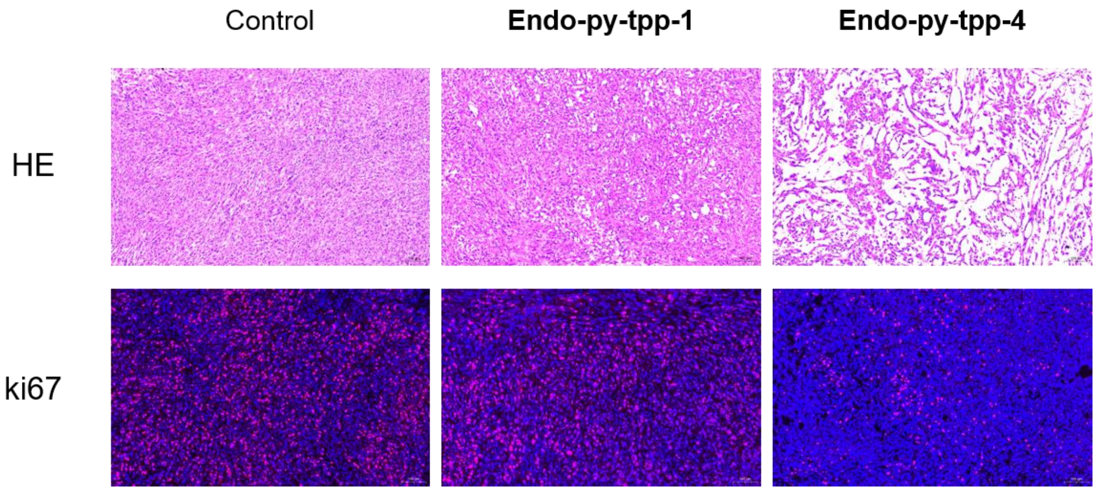

Fig S27. Representative H&E, Ki67 staining images of tumor tissues at each group. Scale bar: 100 μm.

16. NMR Spectra

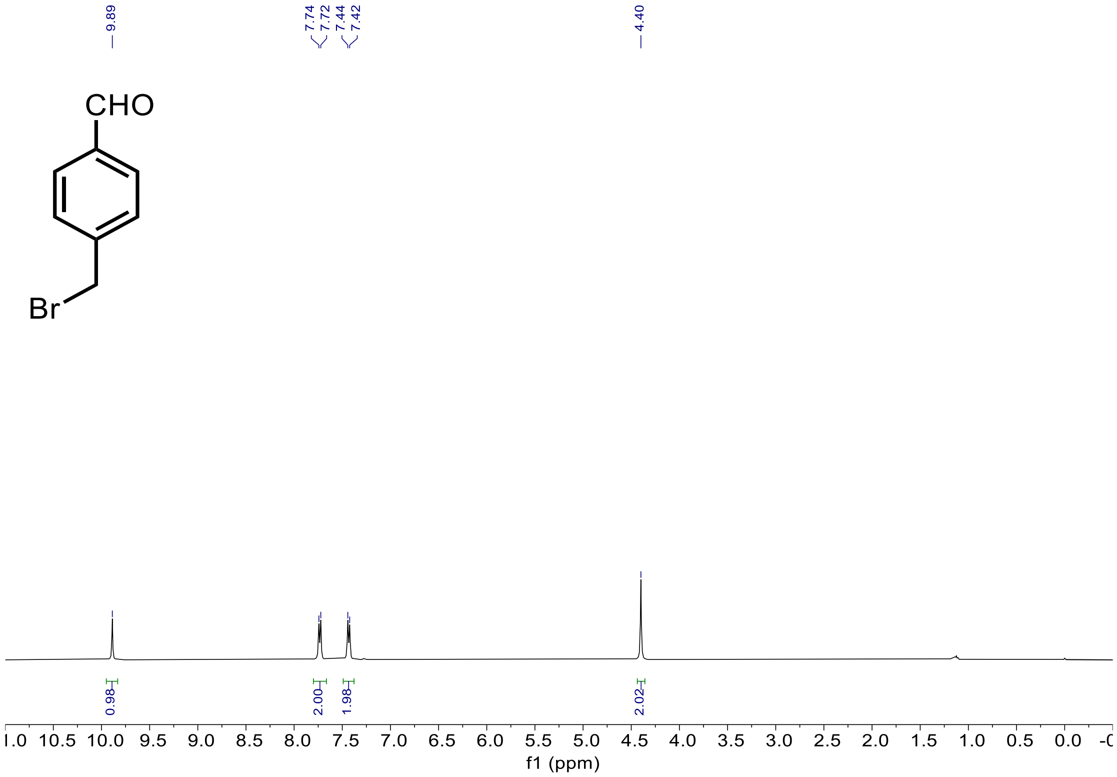

^{1}H NMR of compound **2**

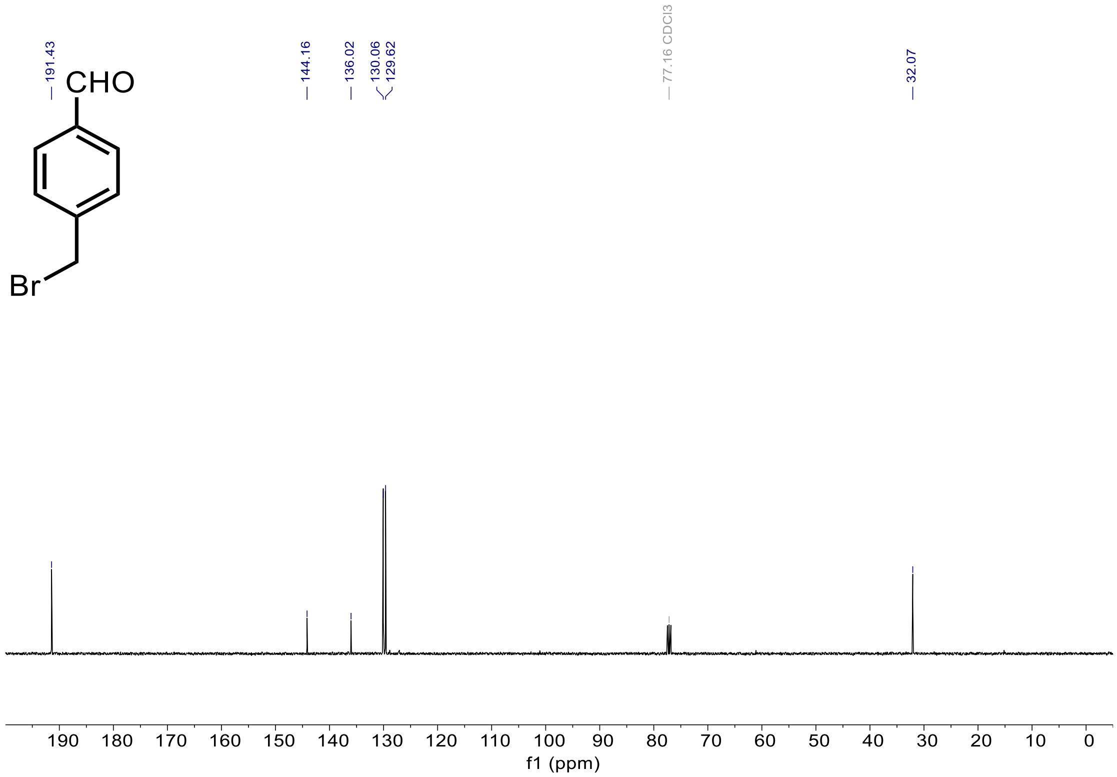

^{13}C NMR of compound **2**

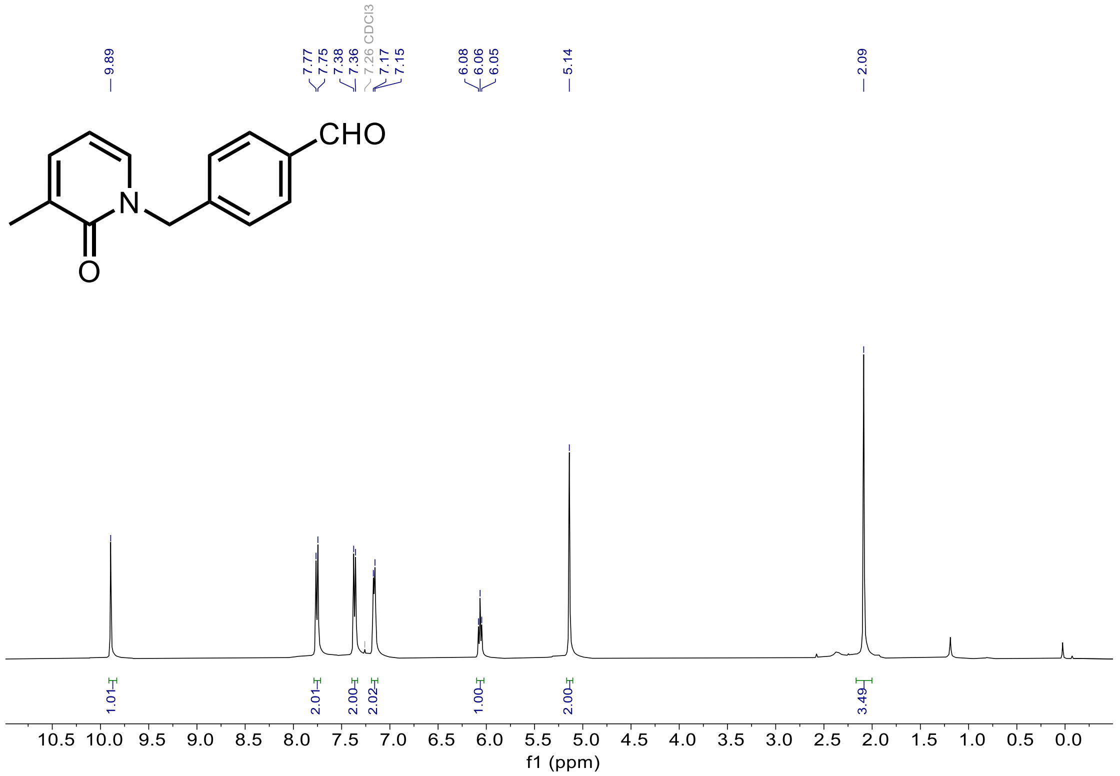

^{1}H NMR of compound **3**

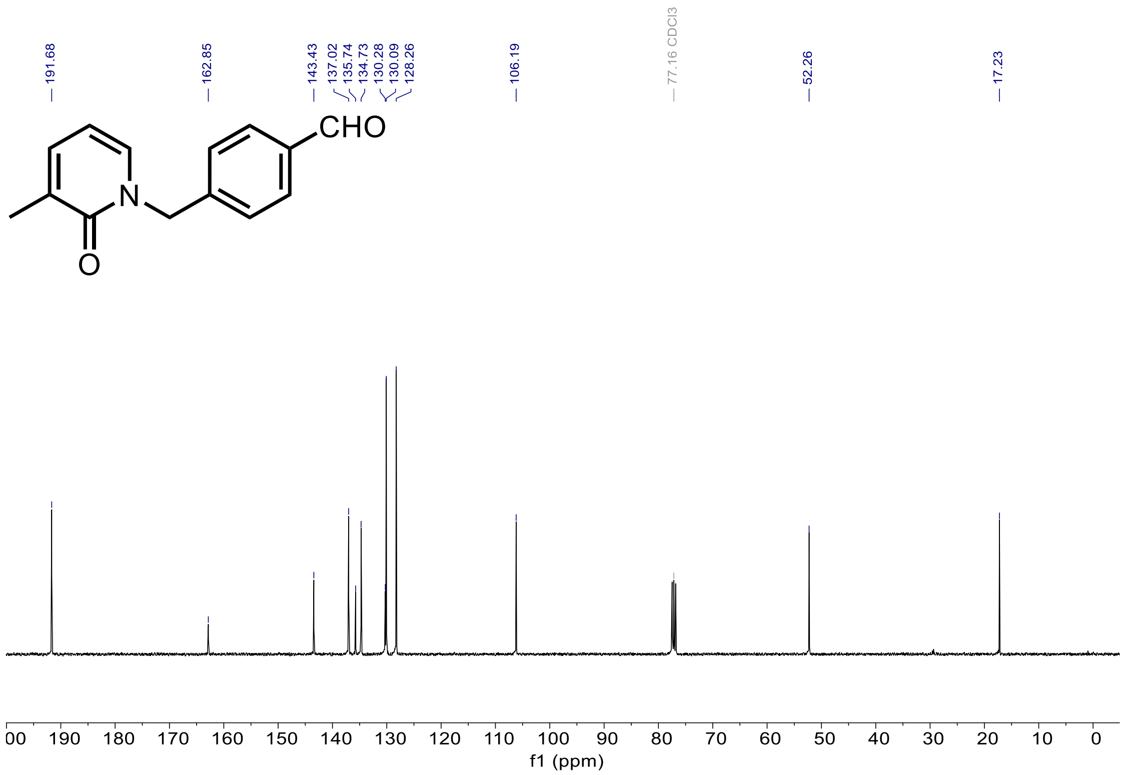

^{13}C NMR of compound **3**

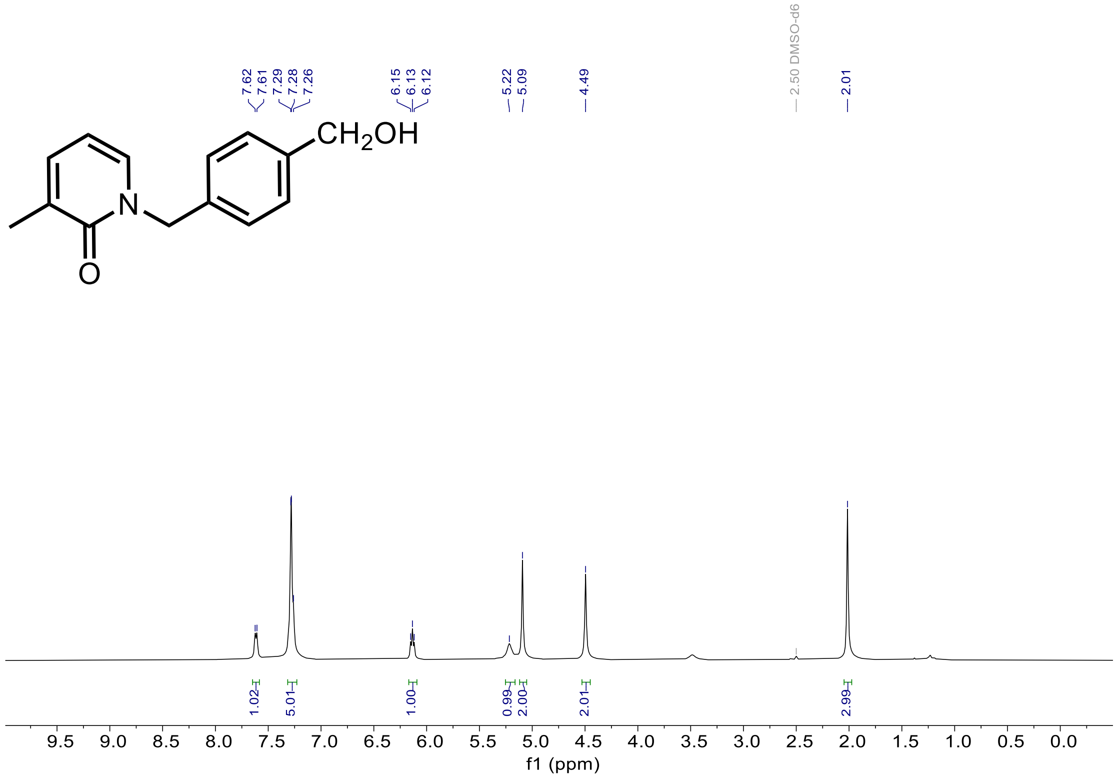

^{1}H NMR of compound **4**

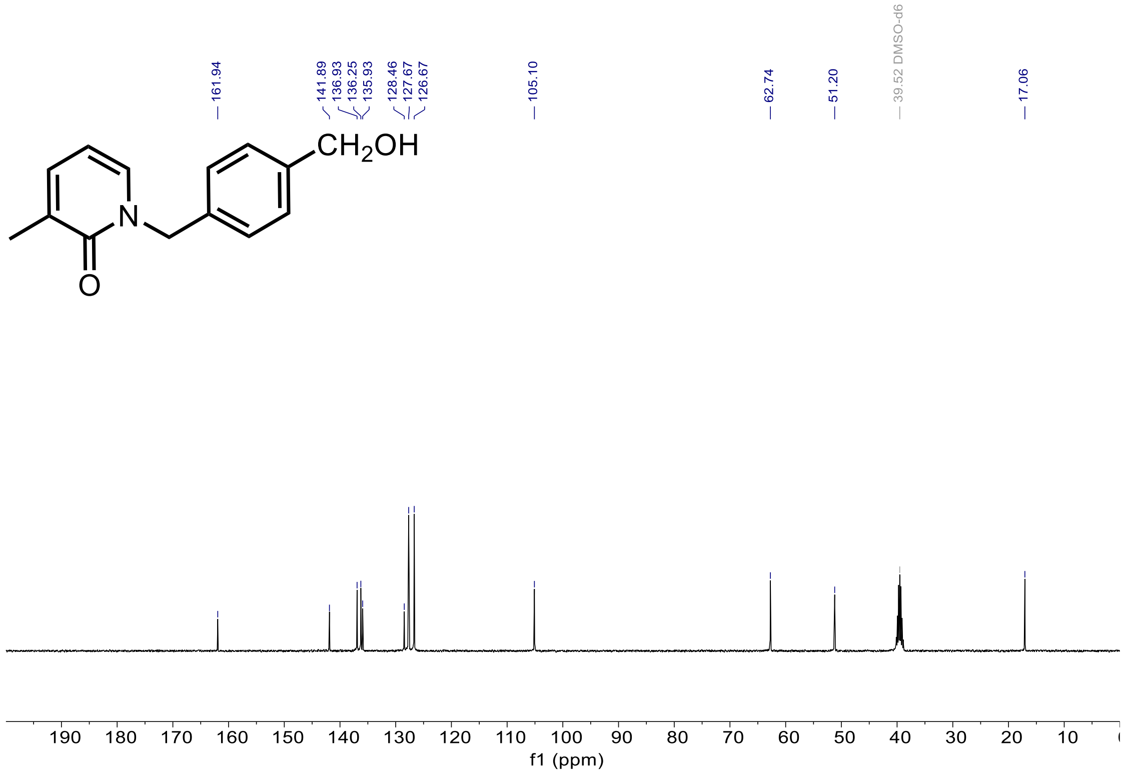

^{13}C NMR of compound **4**

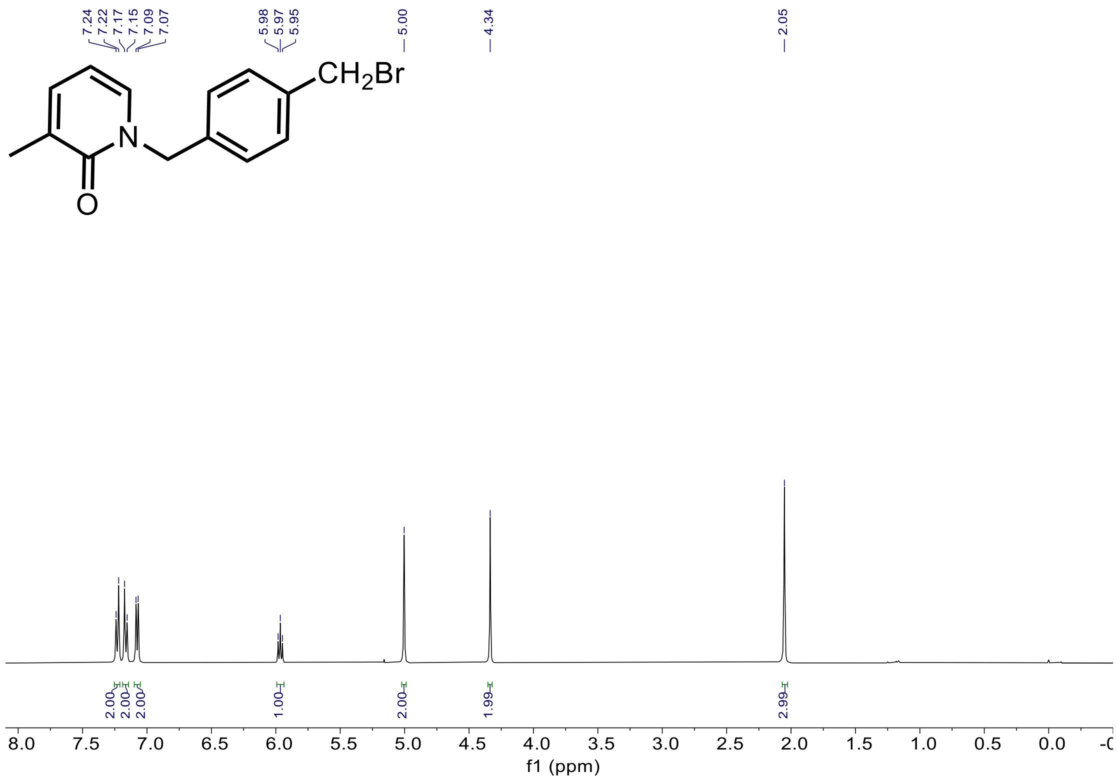

^{1}H NMR of compound **5**

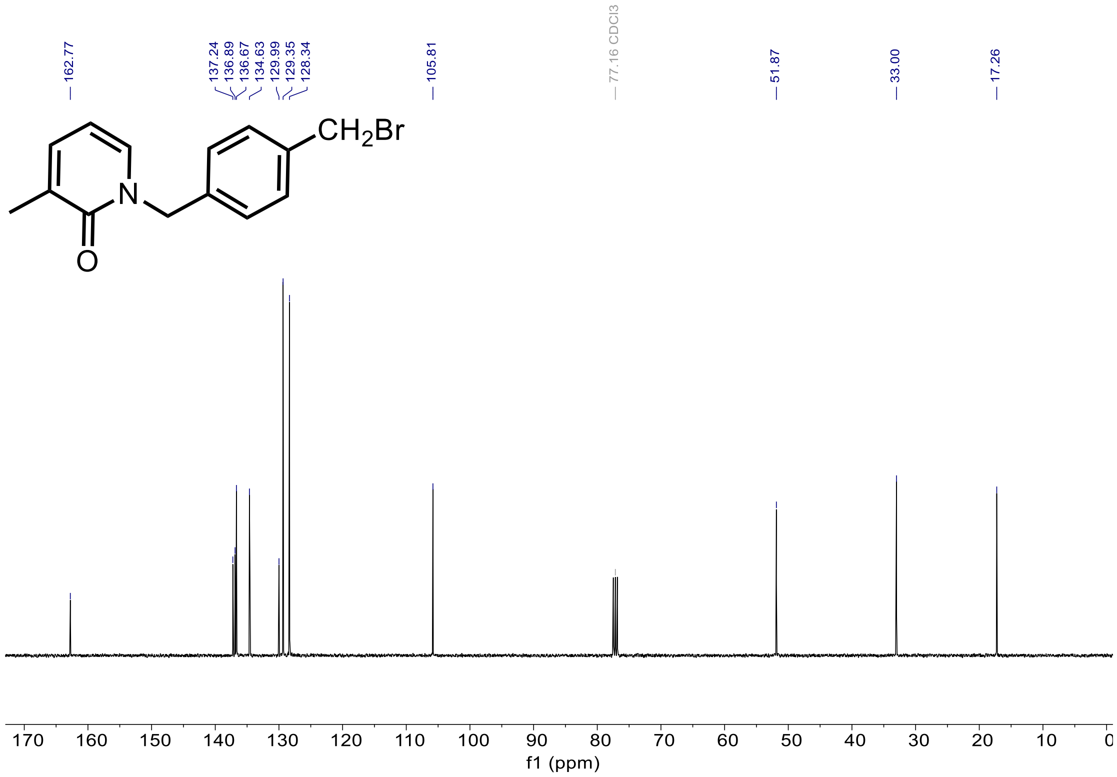

^{13}C NMR of compound **5**

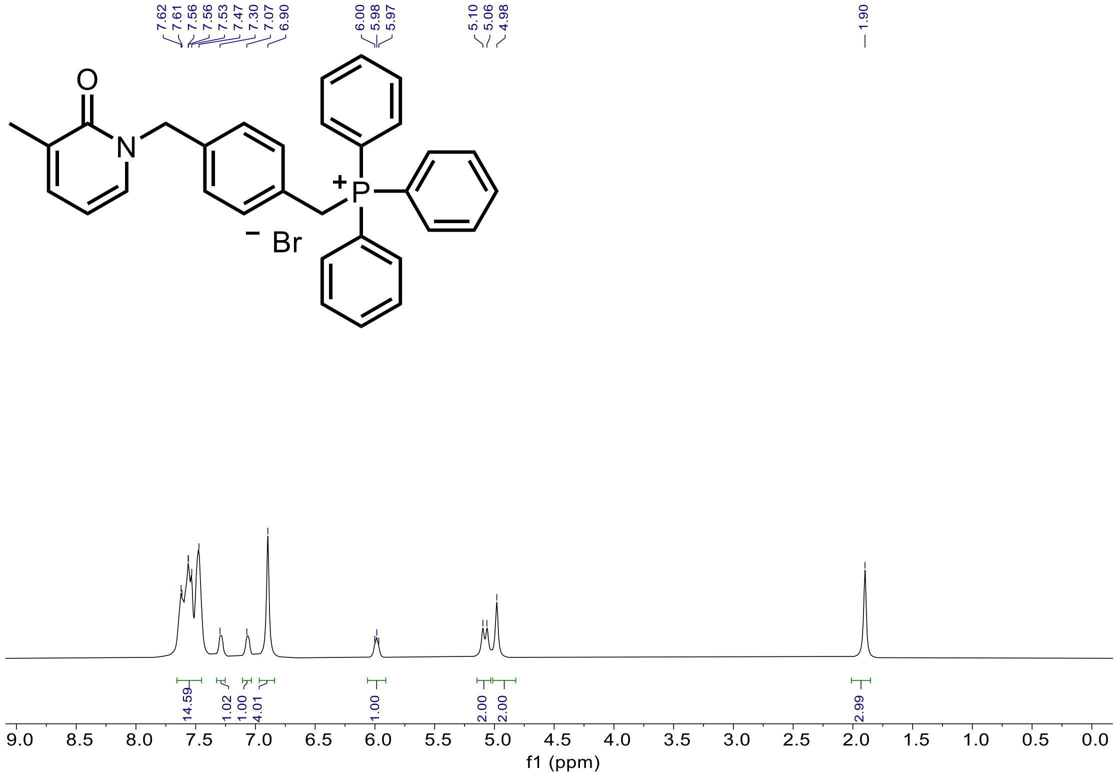

^{1}H NMR of compound **6**

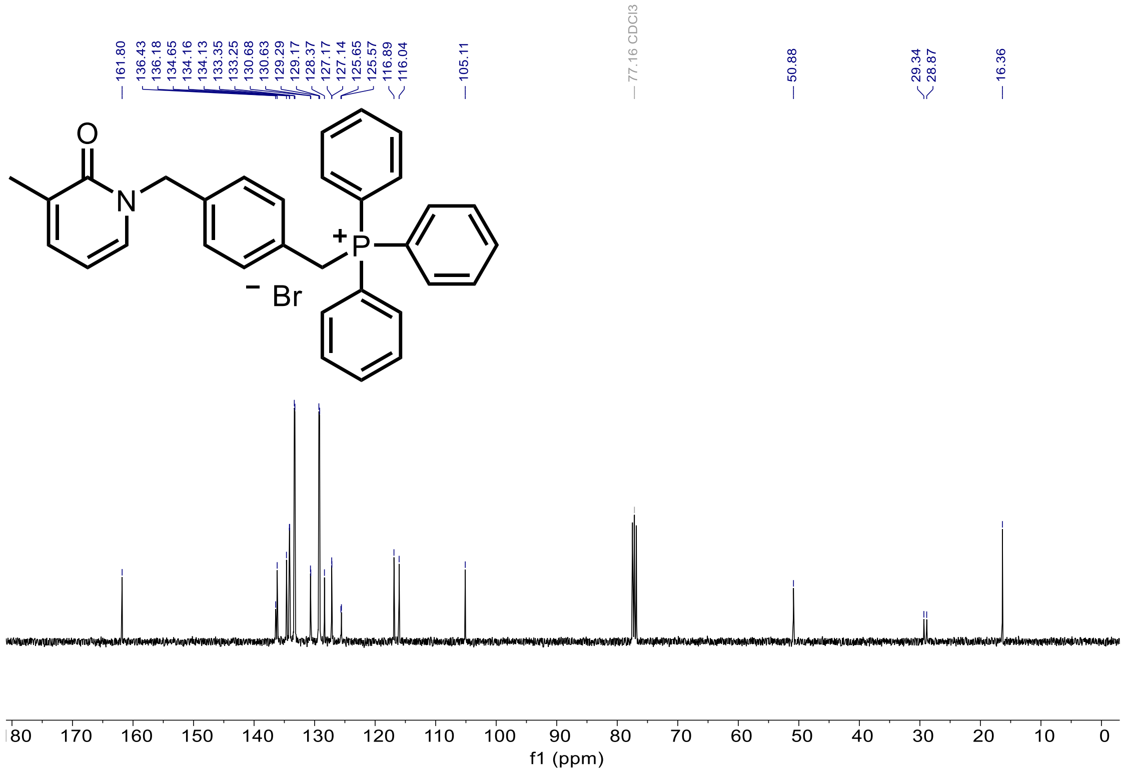

^{13}C NMR of compound **6**

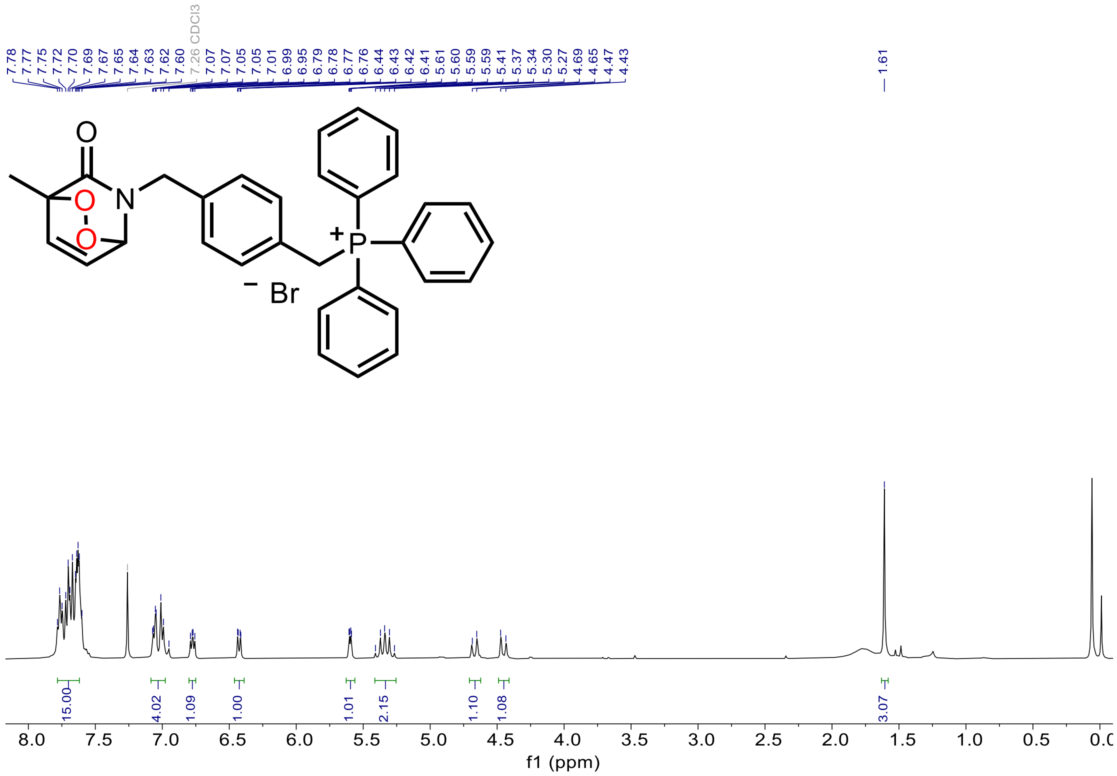

^{1}H NMR of compound **Endo-py-tpp-1**

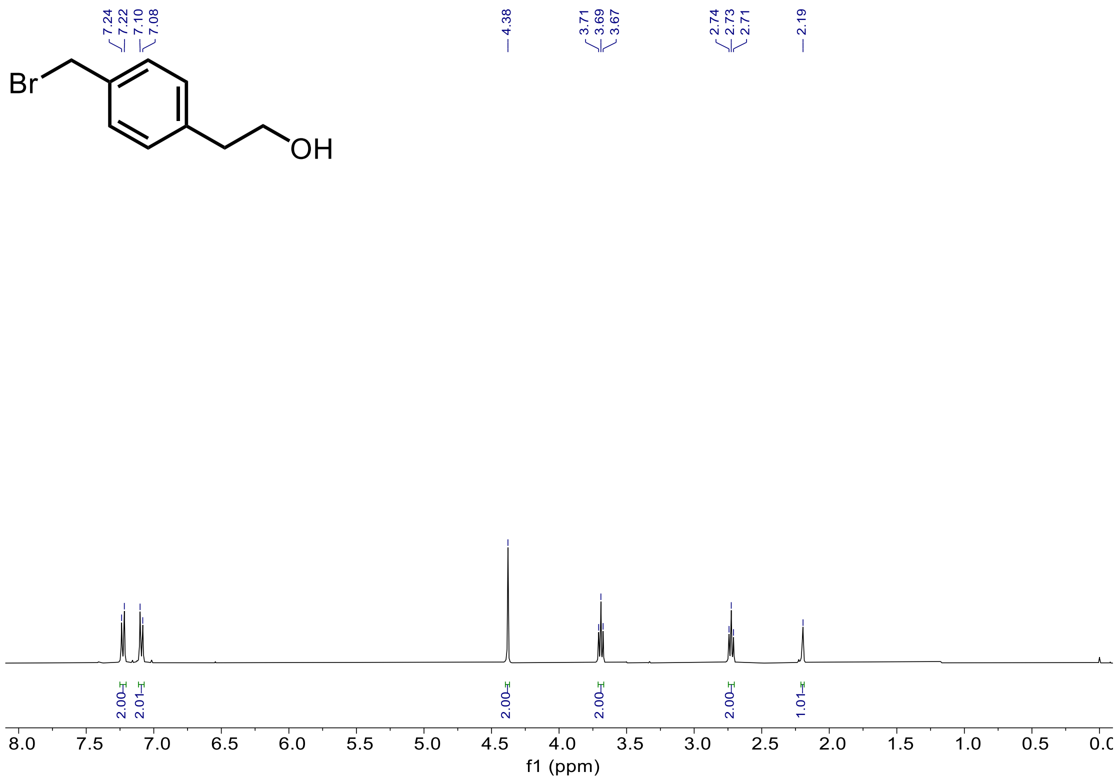

^{1}H NMR of compound **8**

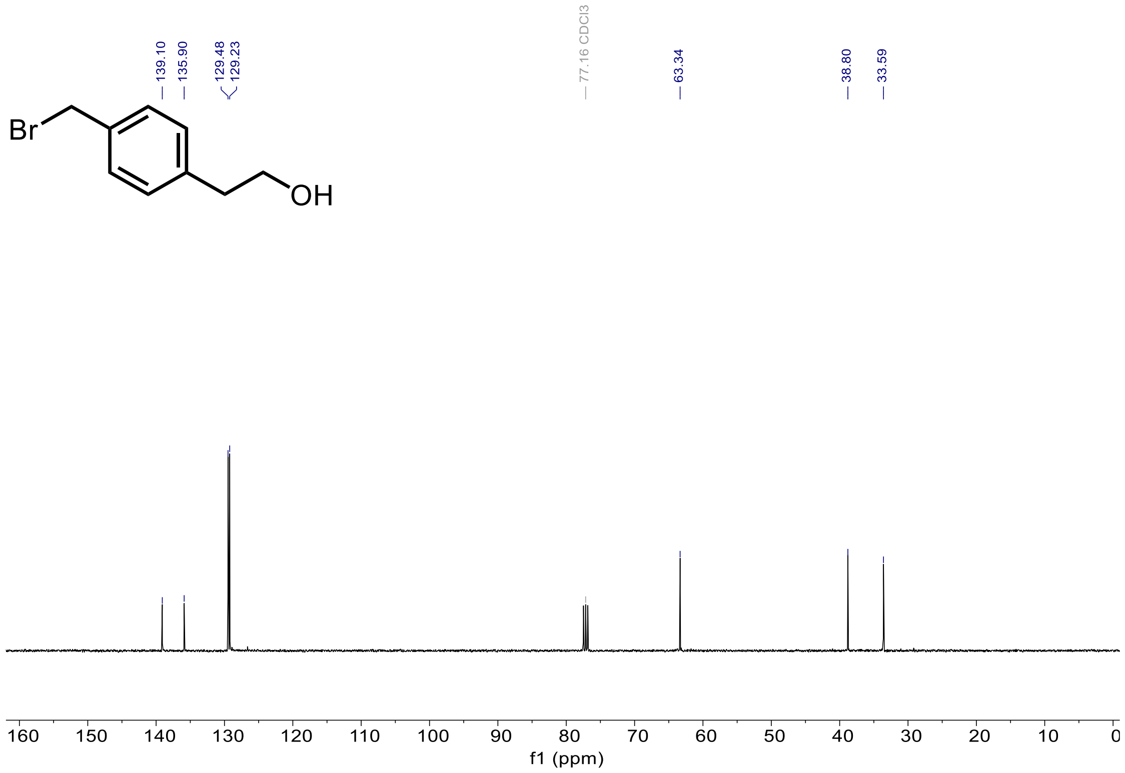

^{13}C NMR of compound **8**

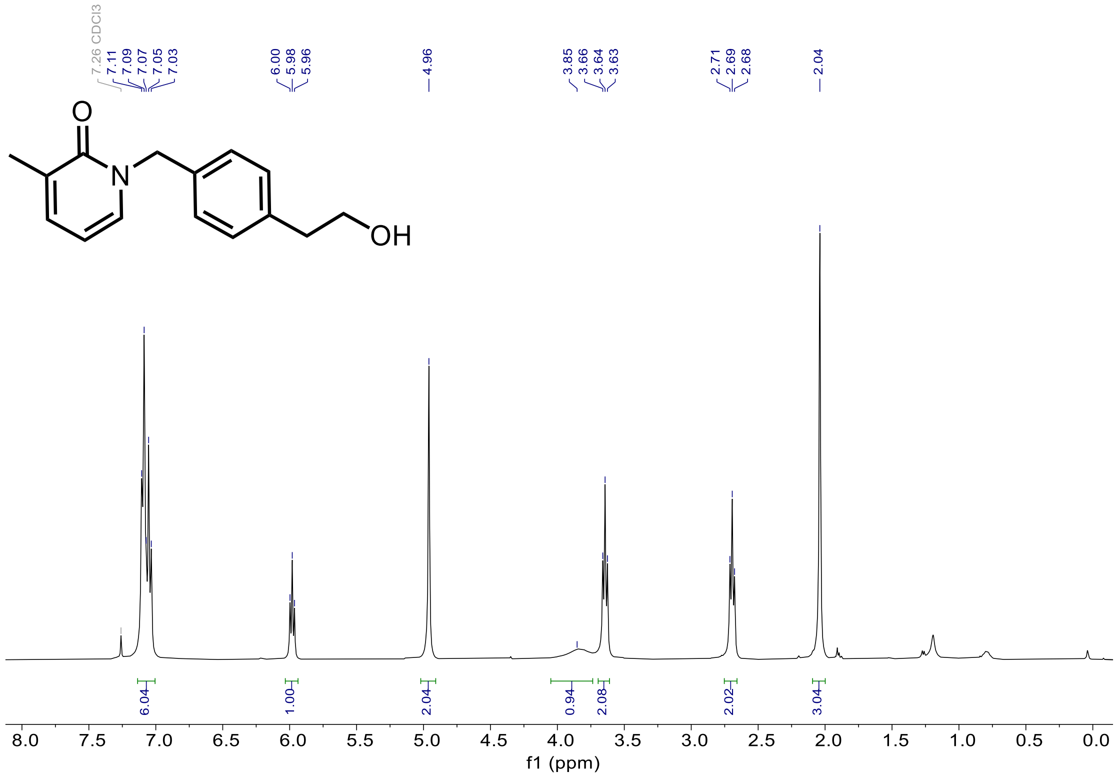

^{1}H NMR of compound **9**

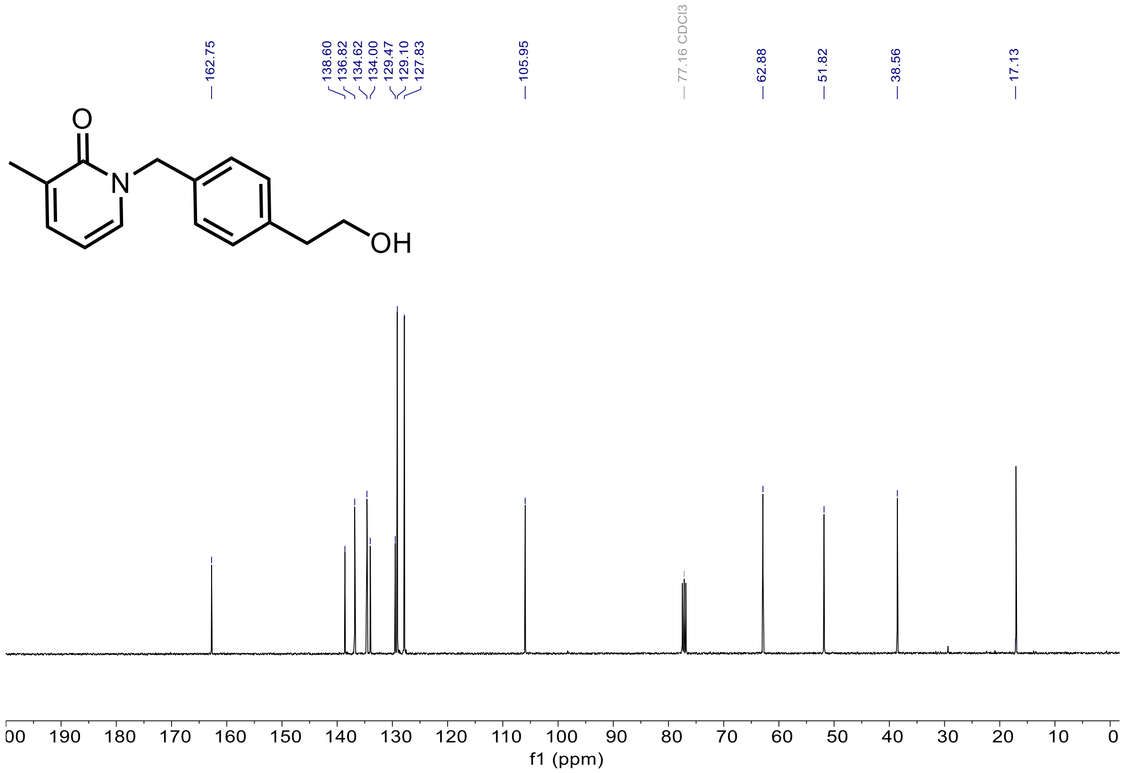

^{13}C NMR of compound **9**

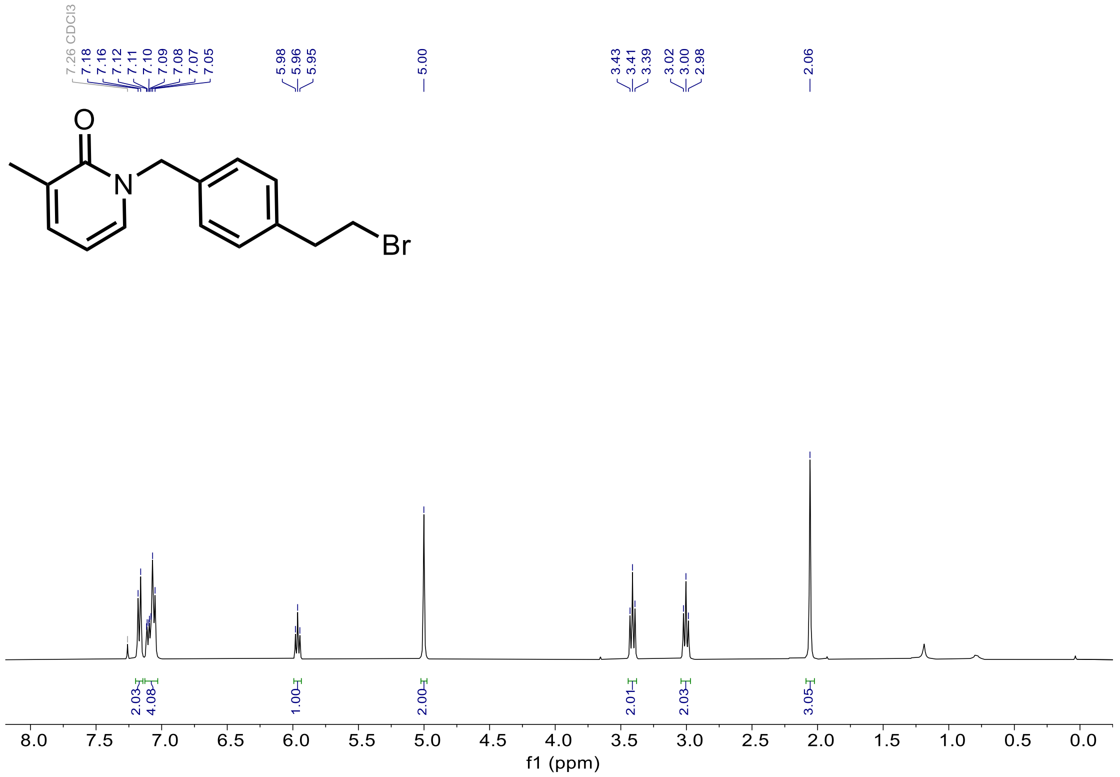

^{1}H NMR of compound **10**

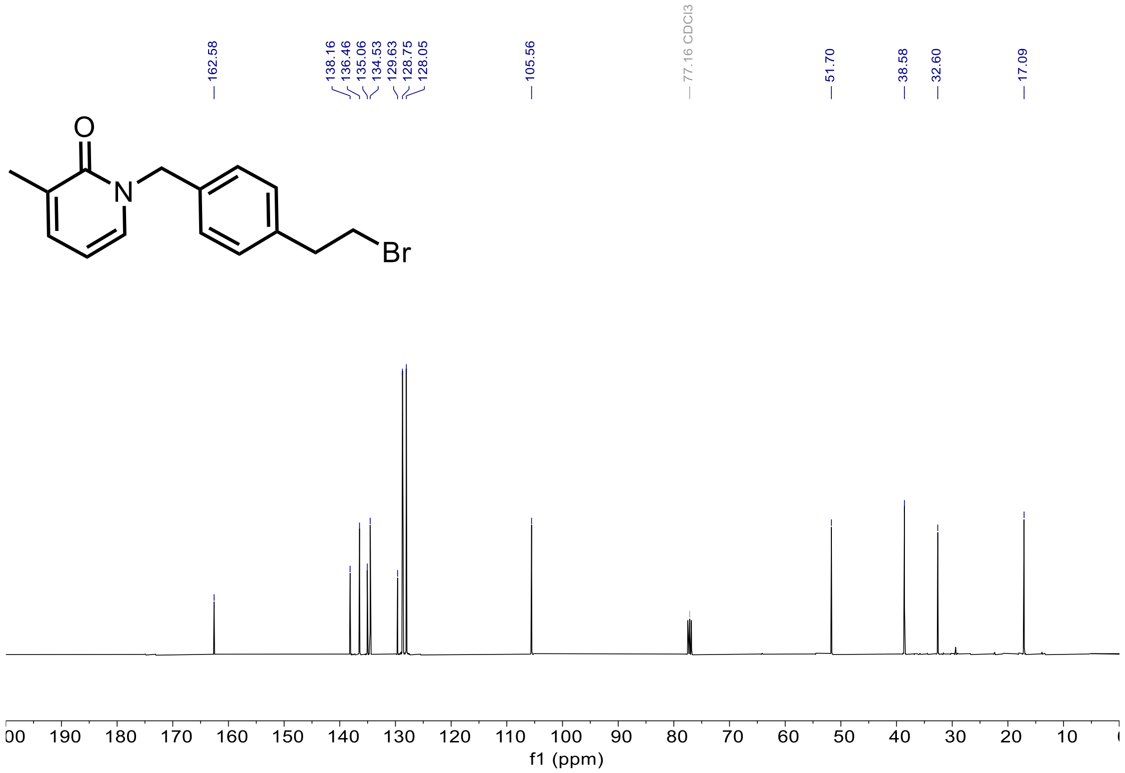

^{13}C NMR of compound **10**

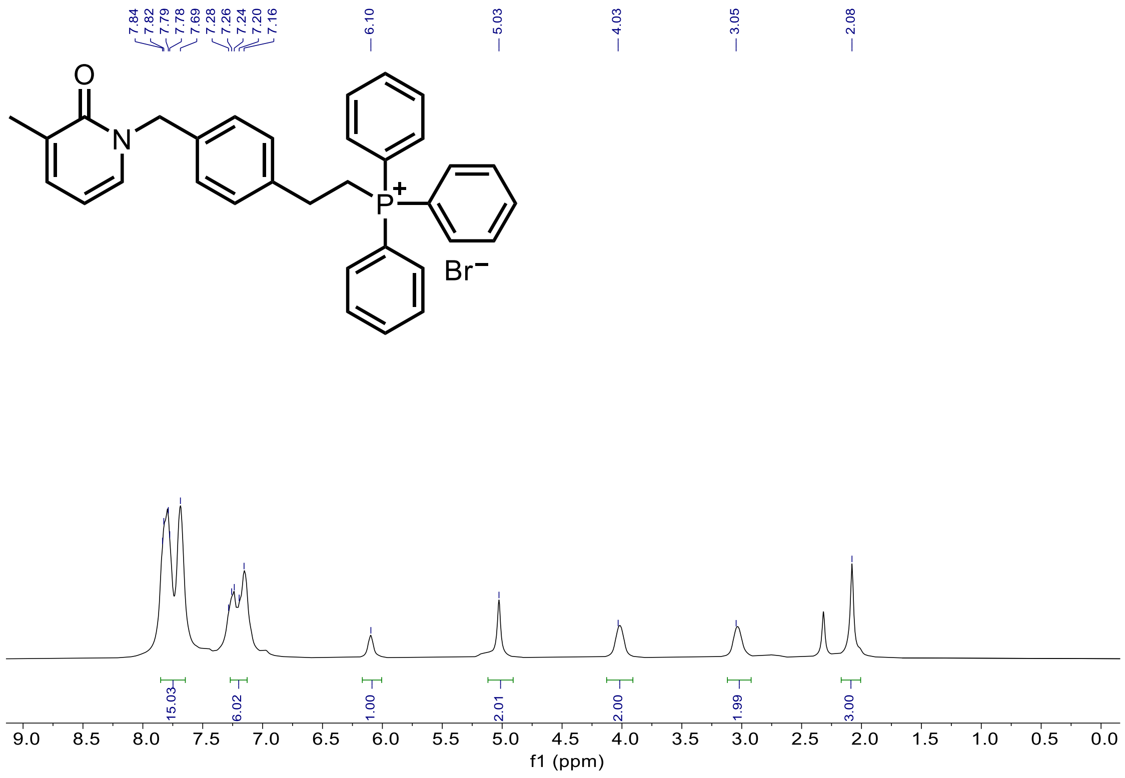

^{1}H NMR of compound **11**

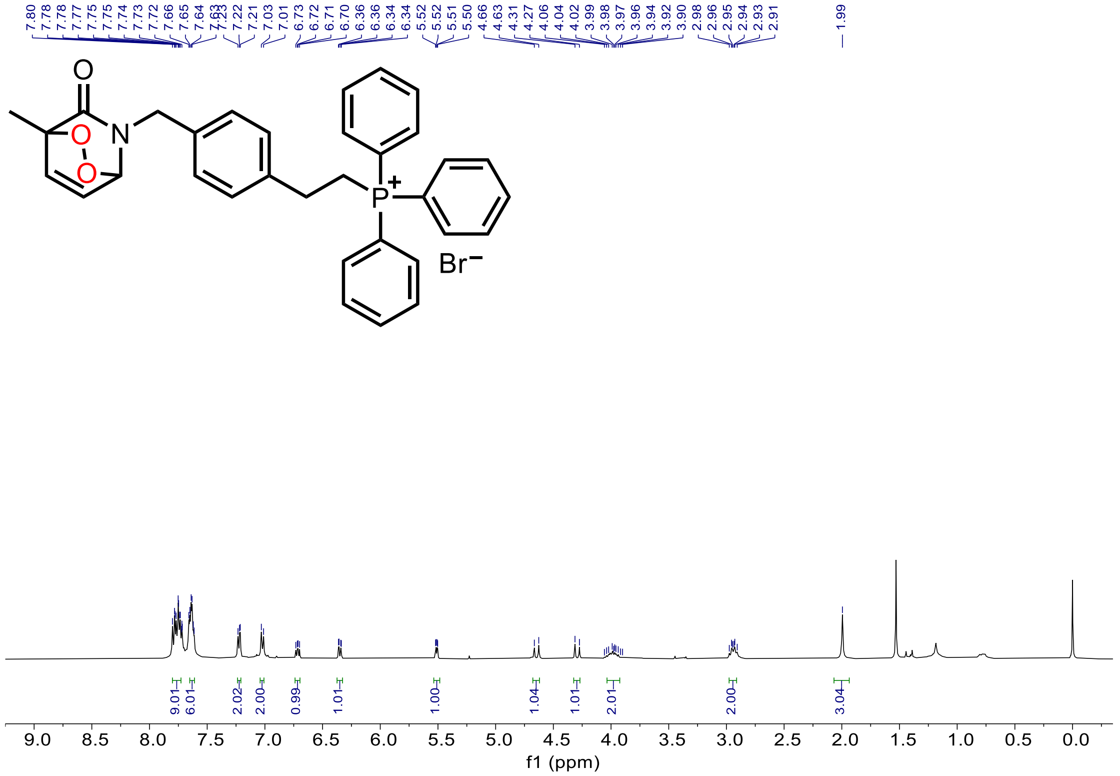

^{1}H NMR of compound **Endo-py-tpp-2**

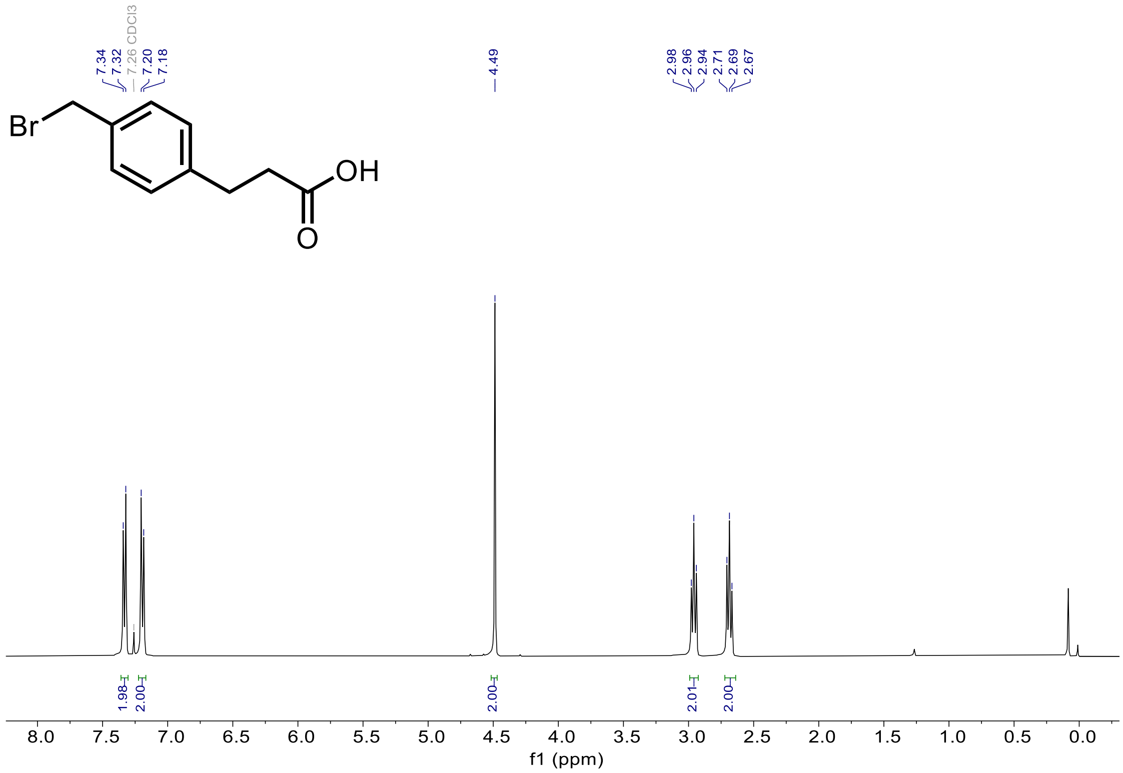

^{1}H NMR of compound **13**

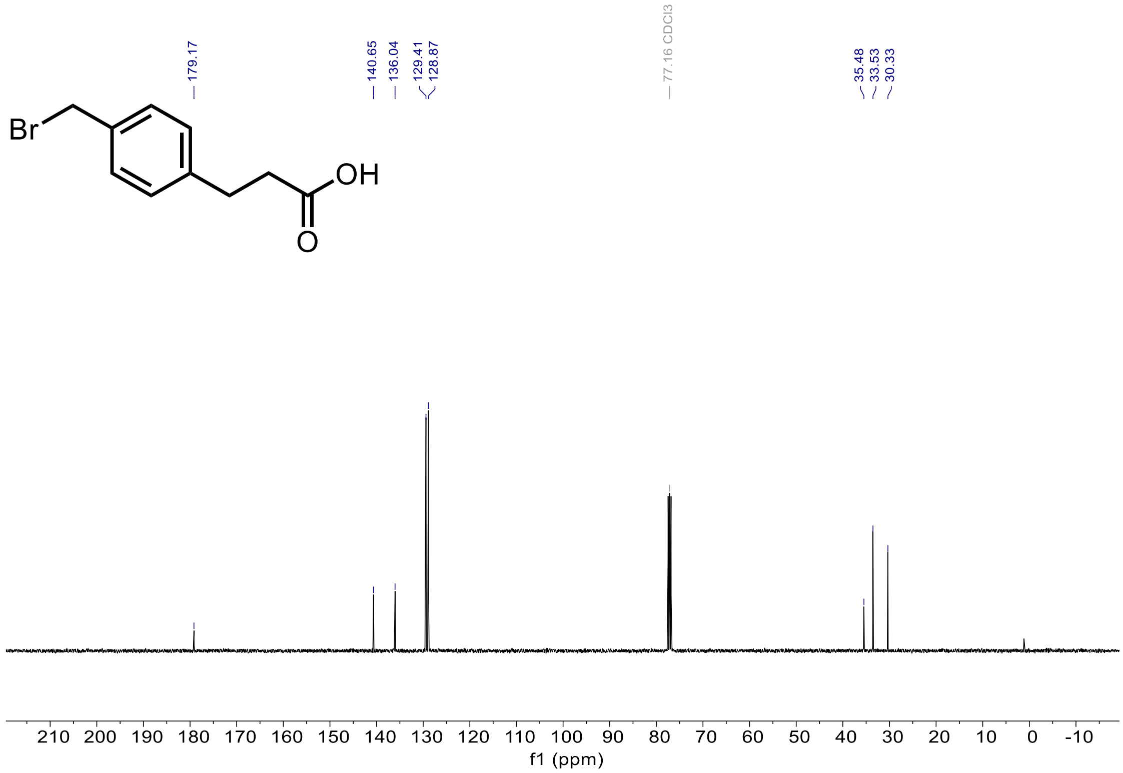

^{13}C NMR of compound **13**

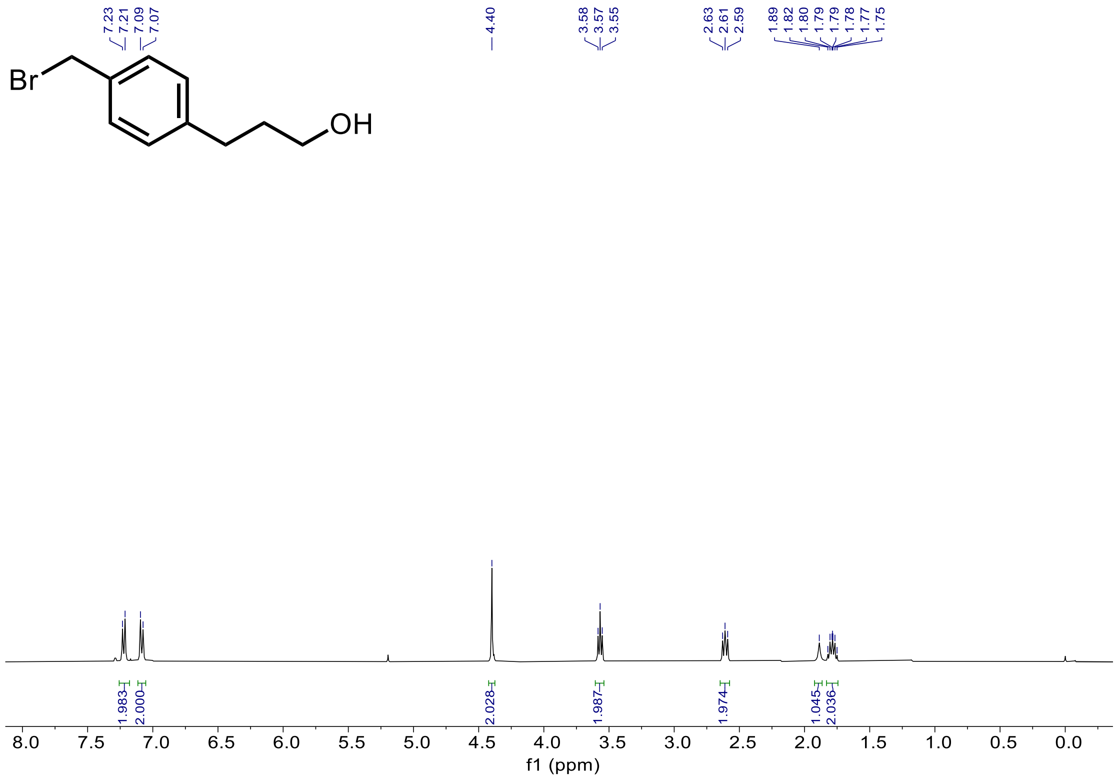

^{1}H NMR of compound **14**

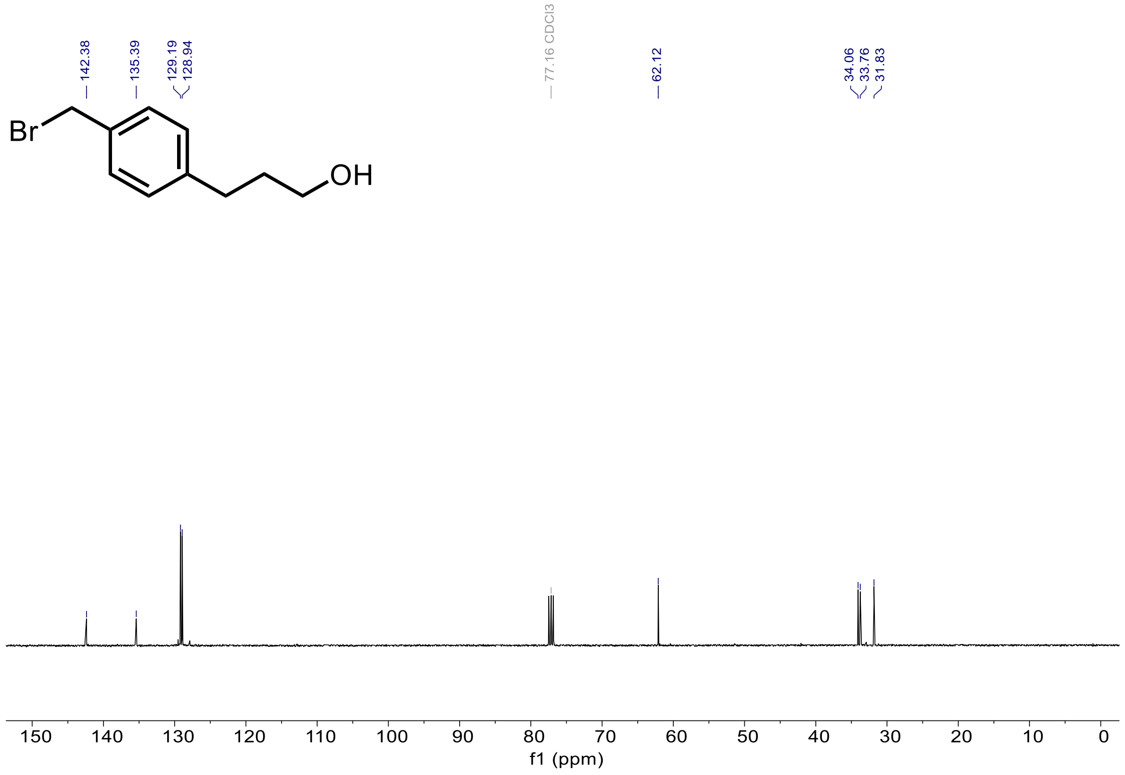

^{13}C NMR of compound **14**

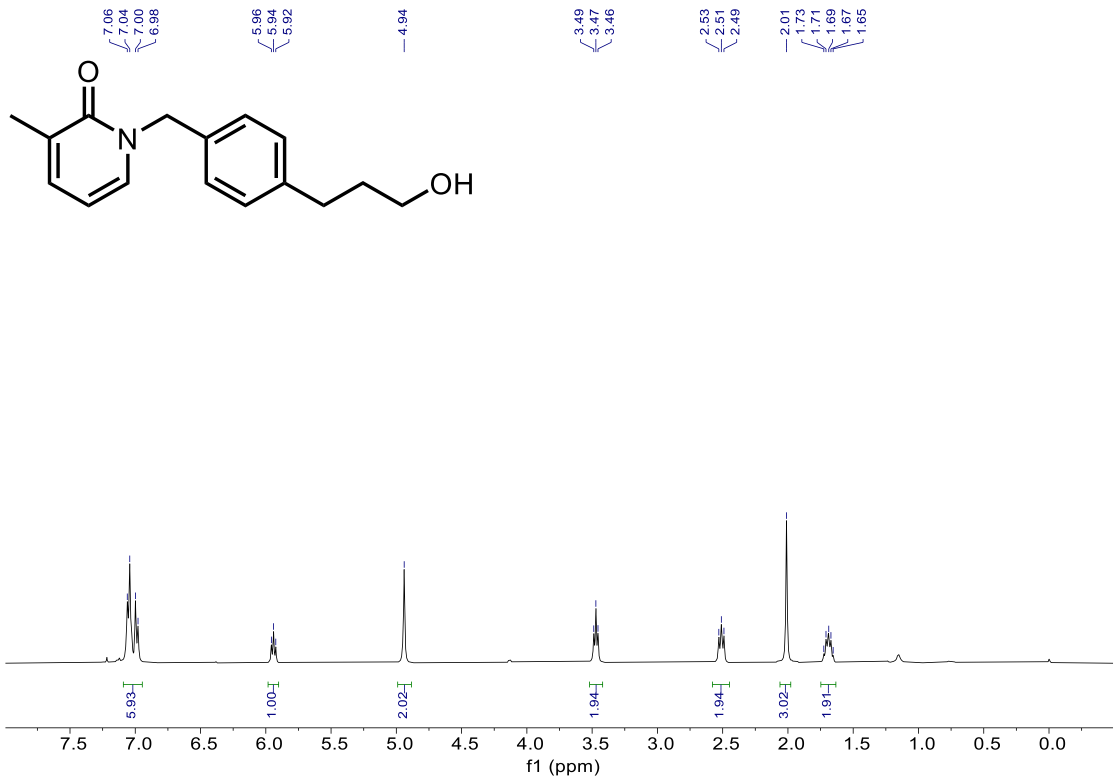

^{1}H NMR of compound **15**

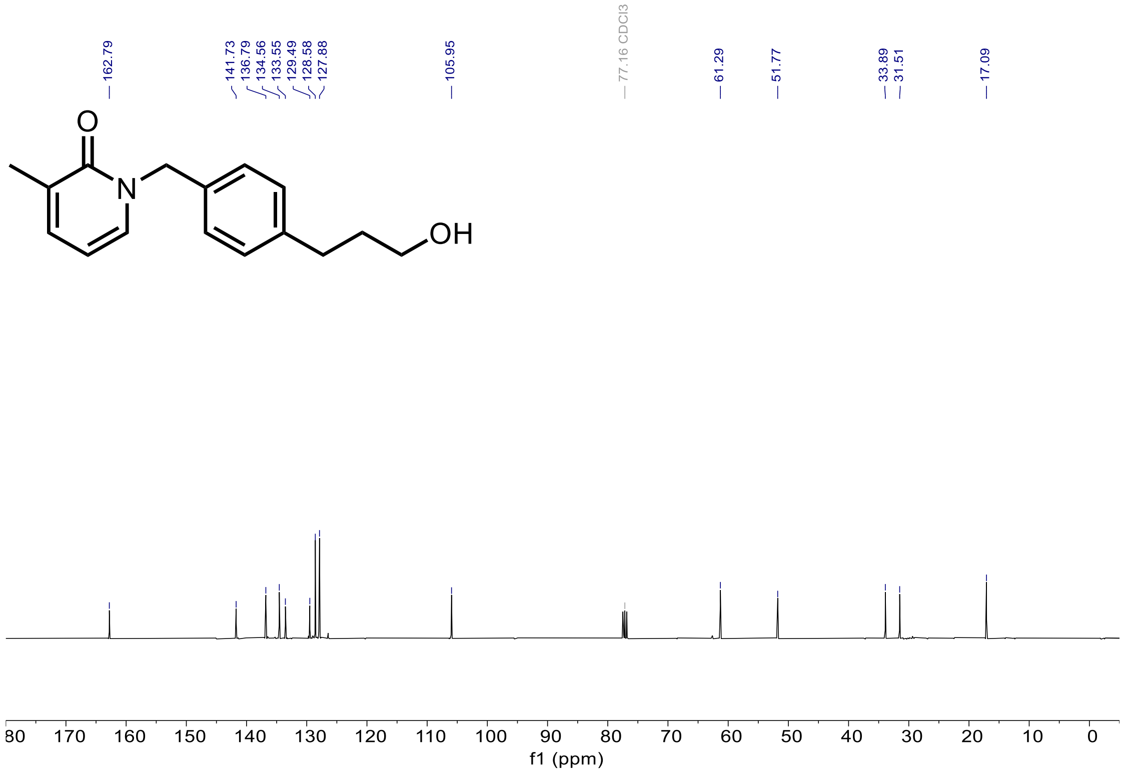

^{13}C NMR of compound **15**

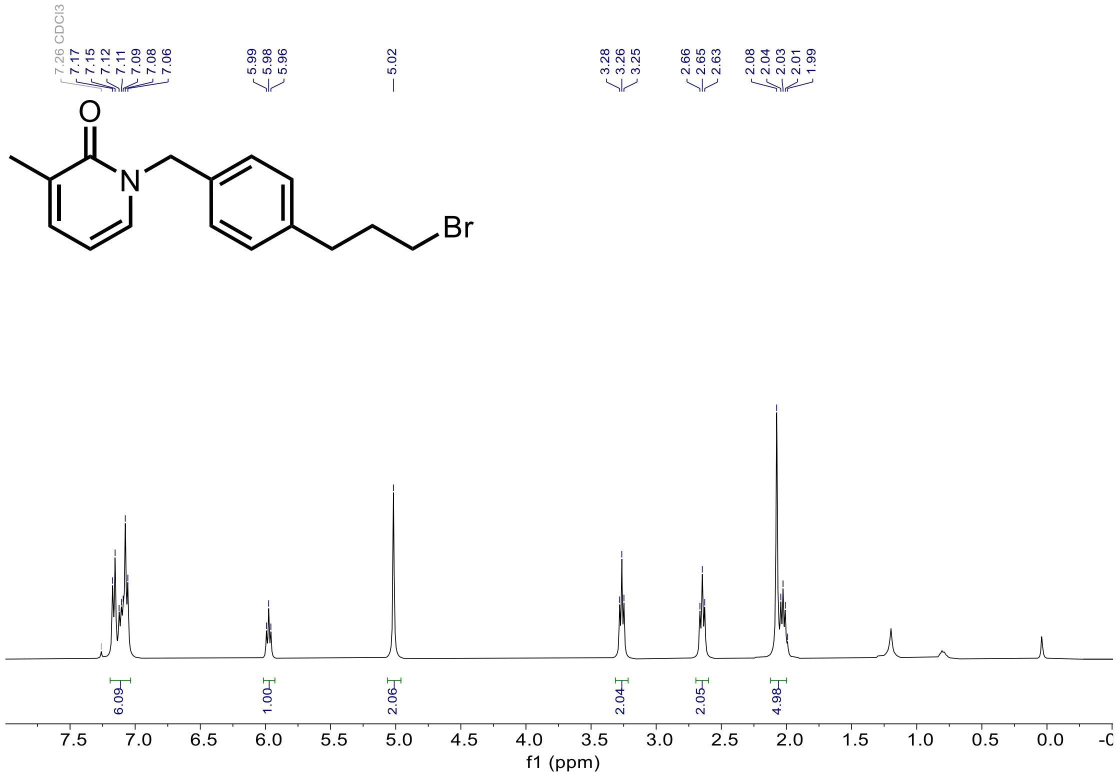

^{1}H NMR of compound **16**

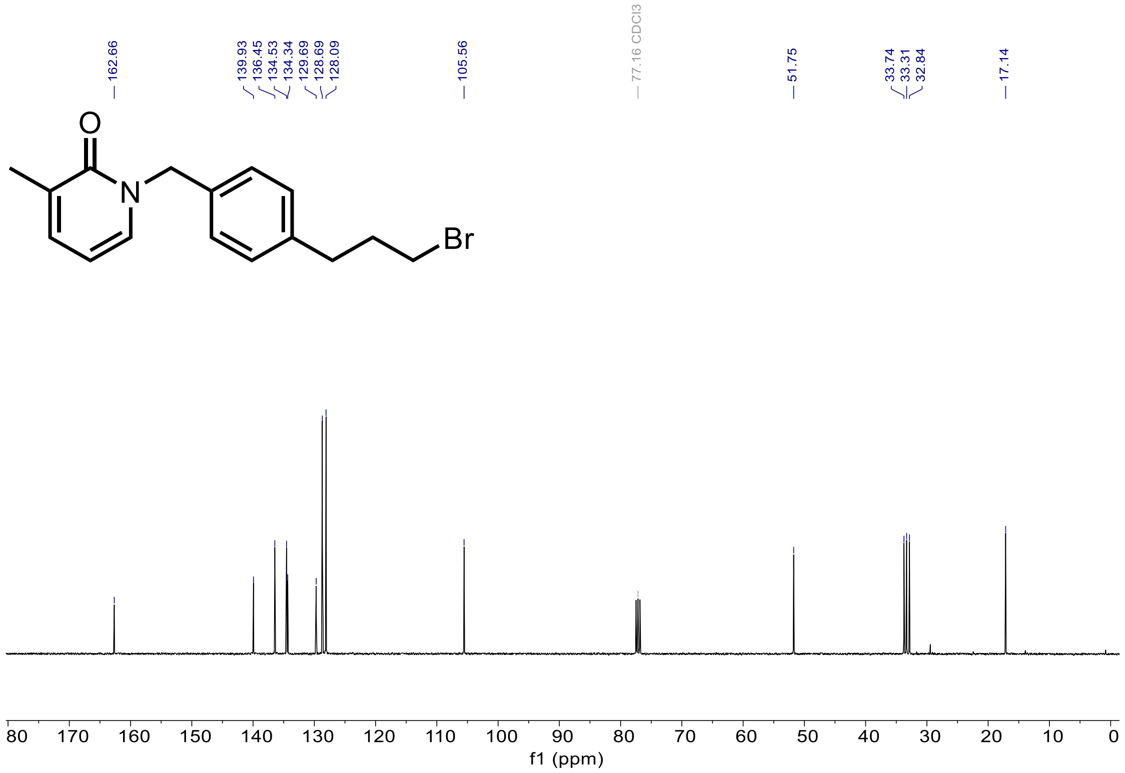

^{13}C NMR of compound **16**

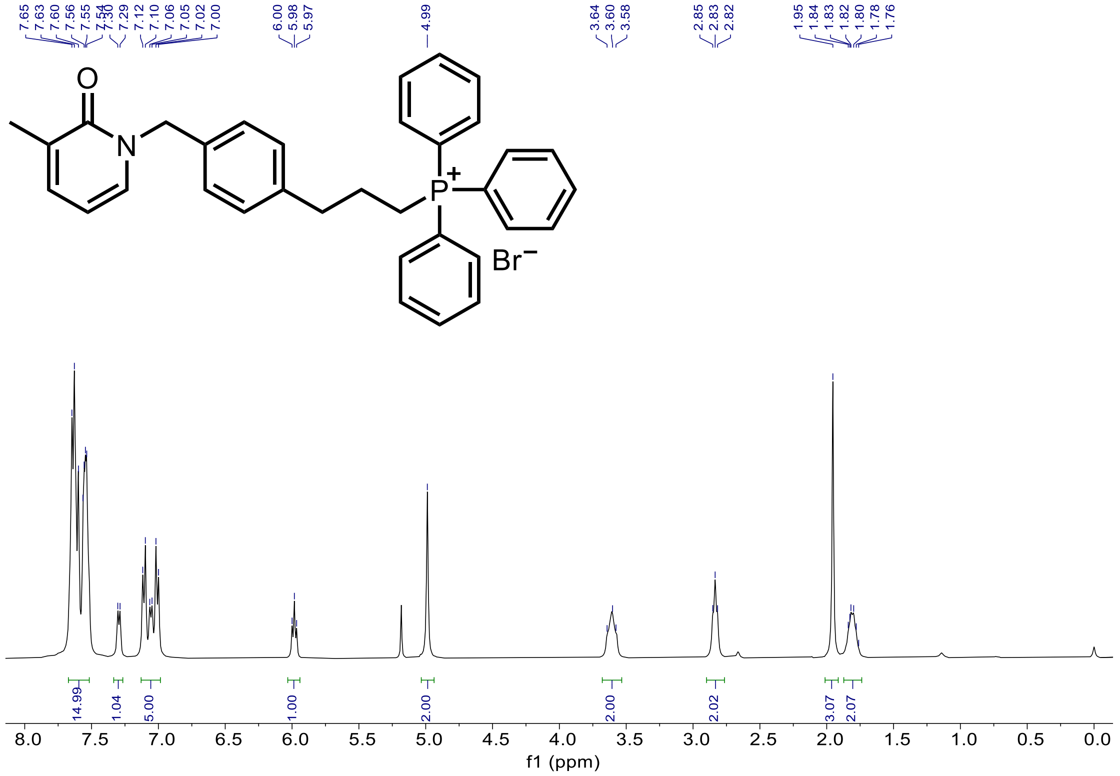

^{1}H NMR of compound **17**

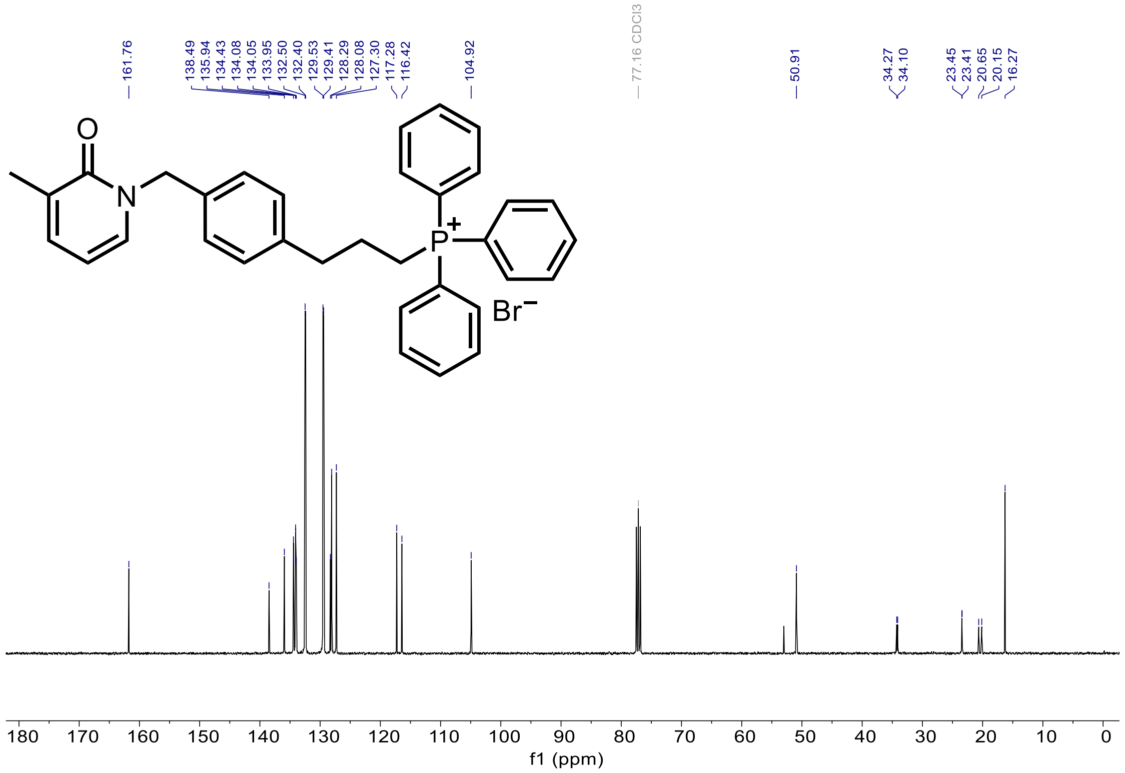

^{13}C NMR of compound **17**

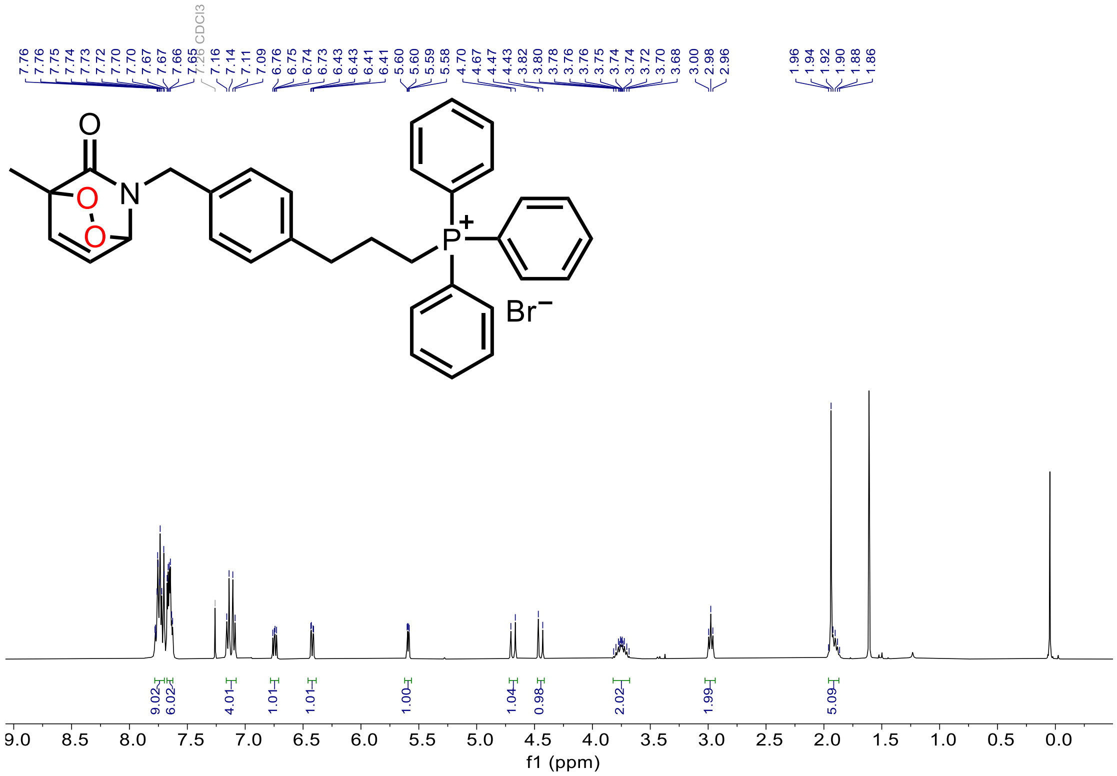

^{1}H NMR of compound **Endo-py-tpp-3**

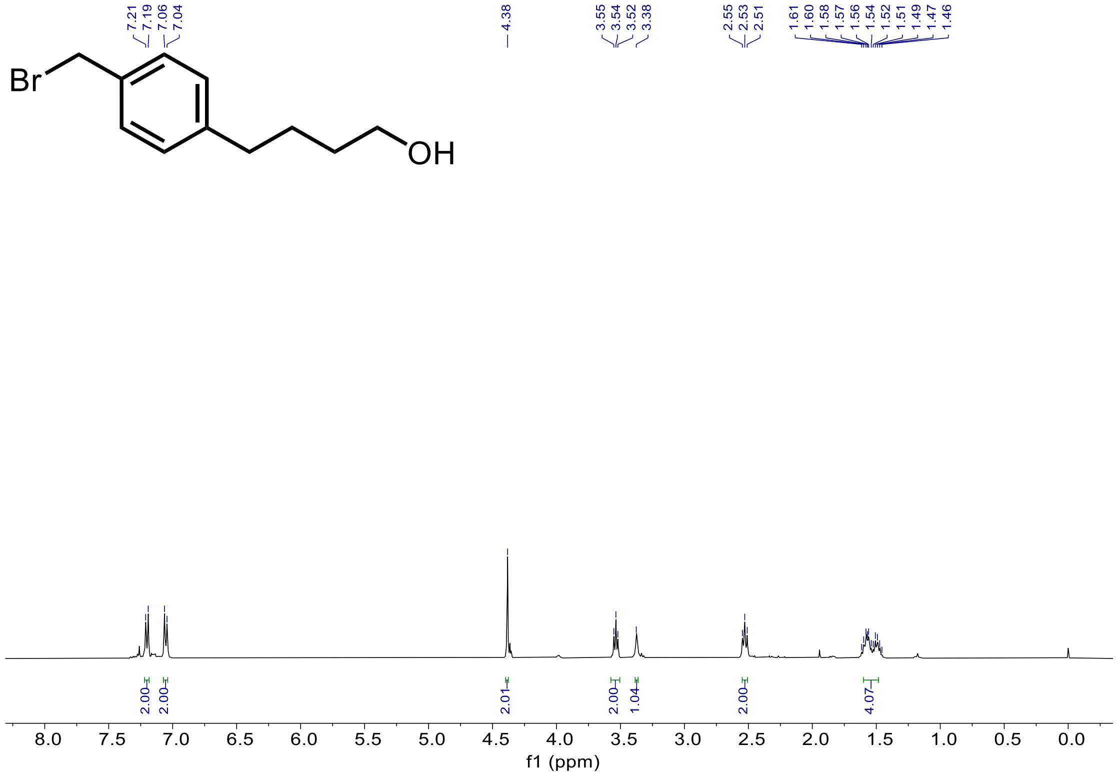

^{1}H NMR of compound **20**

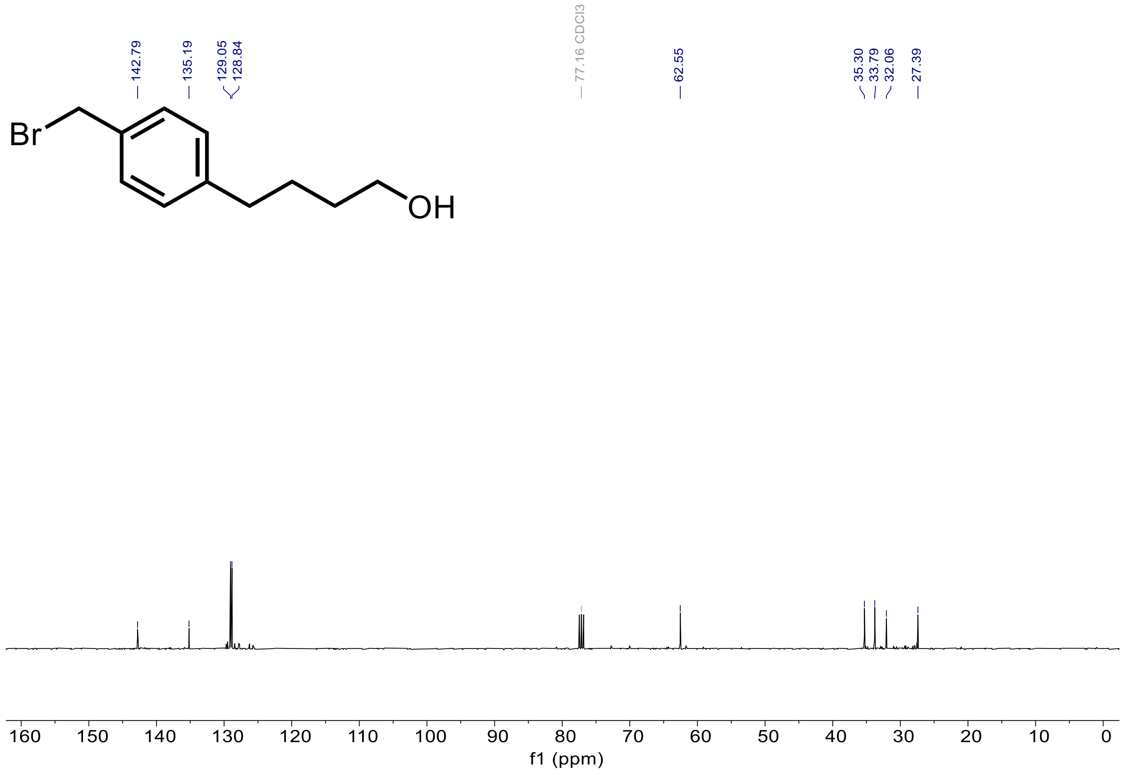

^{13}C NMR of compound **20**

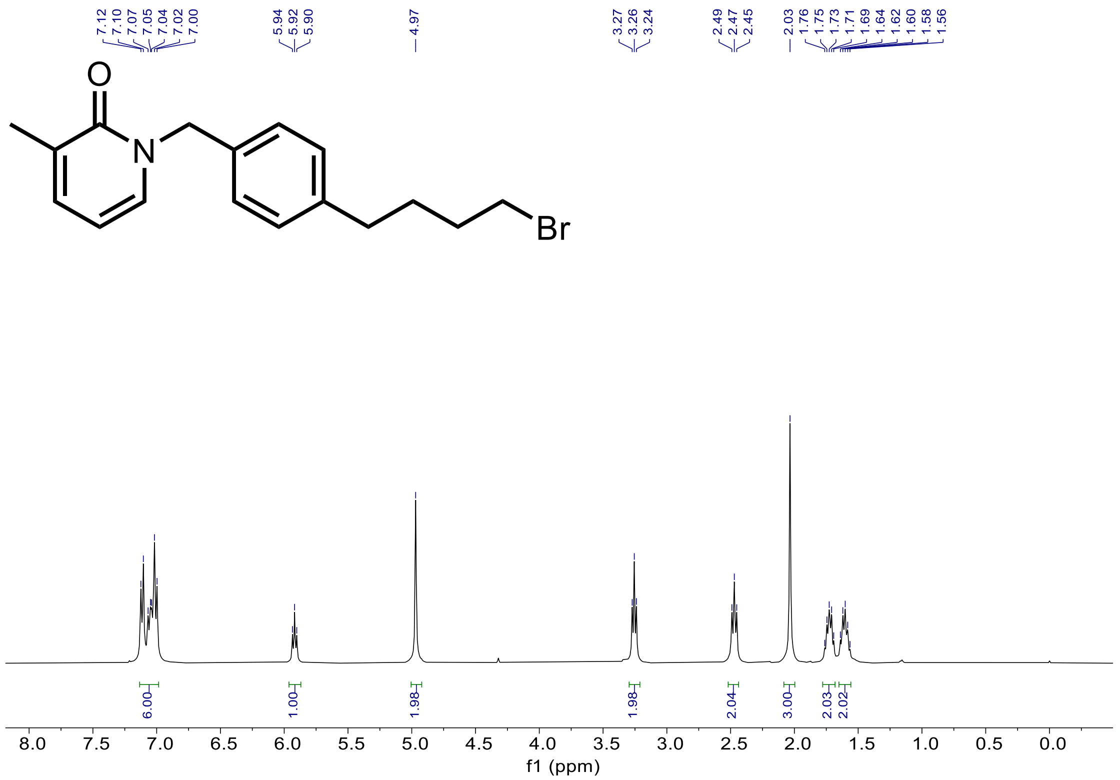

^{1}H NMR of compound **22**

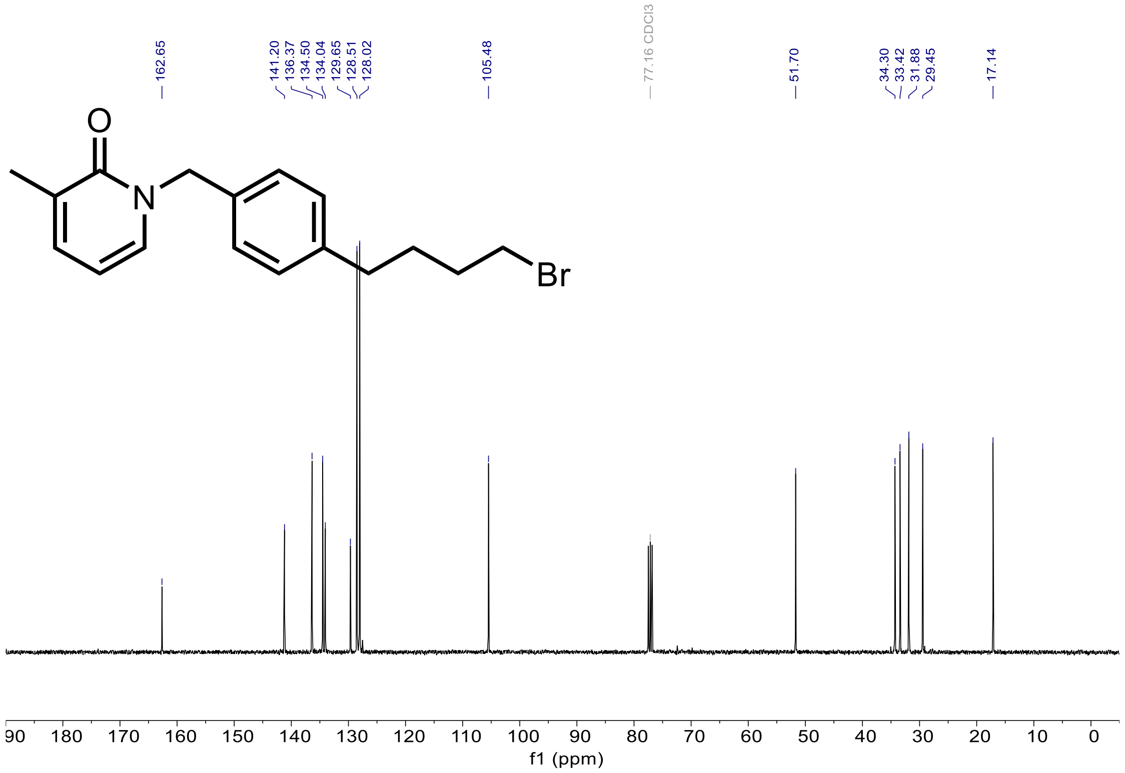

^{13}C NMR of compound **22**

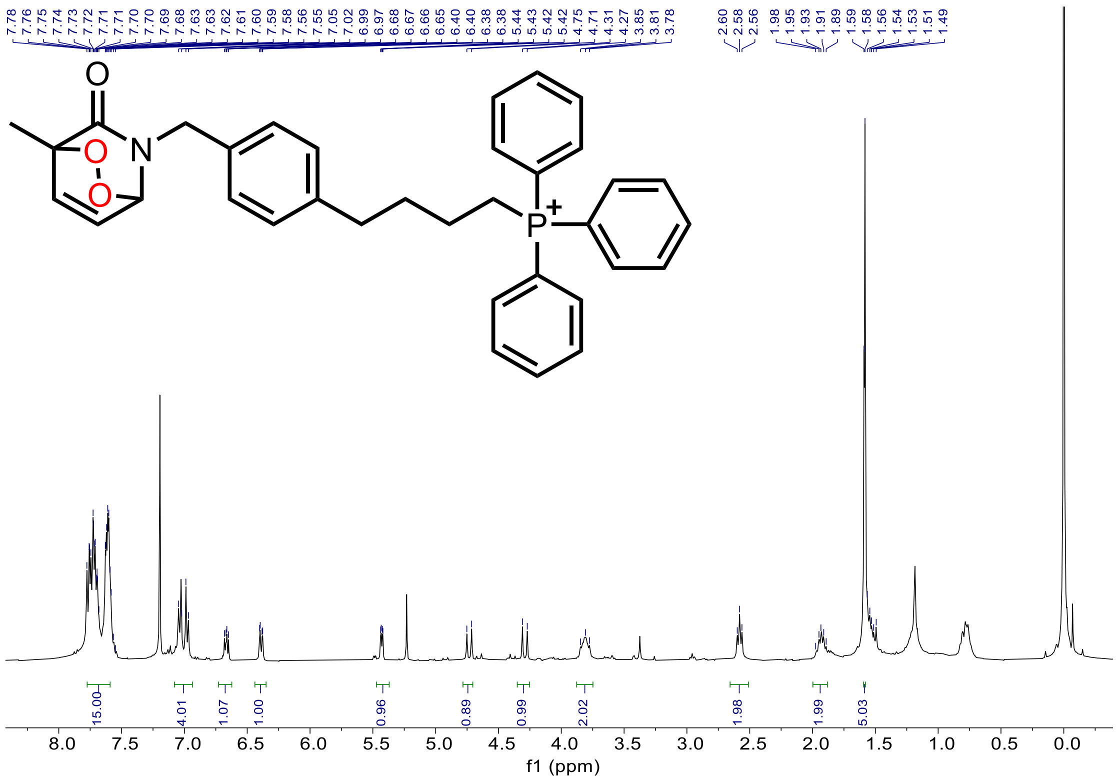

^{1}H NMR of compound **Endo-py-tpp-4**
